# Enhanced therapeutic window for antimicrobial Pept-ins by investigating their structure-activity relationship

**DOI:** 10.1371/journal.pone.0283674

**Published:** 2023-03-31

**Authors:** Guiqin Wu, Laleh Khodaparast, Ladan Khodaparast, Matthias De Vleeschouwer, Nikolaos Louros, Rodrigo Gallardo, Pengpeng Yi, Frederic Rousseau, Joost Schymkowitz

**Affiliations:** 1 VIB-KU Leuven Center for Brain & Disease Research, Leuven, Belgium; 2 Department of Cellular and Molecular Medicine, KU Leuven, Leuven, Belgium; University of Cambridge, UNITED KINGDOM

## Abstract

The overconsumption and inappropriate use of antibiotics is escalating antibiotic resistance development, which is now one of the 10 top threats to global health. Introducing antibiotics with a novel mode of action into clinical use is urgently needed to address this issue. Deliberately inducing aggregation of target proteins and disrupting protein homeostasis in bacteria via amyloidogenic peptides, also called Pept-ins (from *pept*ide *in*terferors), can be lethal to bacteria and shows considerable promise as a novel antibiotic strategy. However, the translation of Pept-ins into the clinic requires further investigation into their mechanism of action and improvement of their therapeutic window. Therefore, we performed systematic structure modifications of 2 previously discovered Pept-ins, resulting in 179 derivatives, and investigated the corresponding impact on antimicrobial potency, cellular accumulation, and ability to induce protein aggregation in bacteria, *in vitro* aggregation property, and toxicity on mammalian cells. Our results show that both Pept-in accumulation and aggregation of target proteins in bacteria are requisite for Pept-in mediated antimicrobial activity. Improvement of these two parameters can be achieved via increasing the number of arginine residues, increasing Pept-in aggregation propensity, optimizing the aggregate core structure, adopting β-turn linkers, or forming a disulphide bond. Correspondingly, improvement of these two parameters can enhance Pept-in antimicrobial efficacy against wildtype *E*. *coli* BL21 used in the laboratory as well as clinically isolated multidrug-resistant strain *E*. *coli* ATCC, *A*. *baumannii*, and *K*. *pneumoniae*.

## Introduction

Antibiotic resistance is an emerging global health issue, contributing to increased healthcare costs and increased in-hospital mortality [1, 2]. The overuse of antibiotics and the ability of bacteria to quickly acquire resistance mechanisms have caused the rapid evolvement of antibiotic resistance [3, 4]. The development of novel antibiotics, which can delay the occurrence of resistance, is urgently required to address the emerging antibiotic resistance crisis and prevent potential pandemics. We have previously demonstrated a promising antibacterial strategy, with a limited probability of resistance development, achieved by inducing widespread aggregation of bacterial proteins and subsequently disrupting bacterial proteostasis using synthetic amyloidogenic peptides (also called Pept-in, from *pept*ide *in*terferors) [5, 6].

Protein aggregation is a sequence-specific mode of self-interaction whereby misfolded proteins polymerise into amorphous aggregates or amyloid fibrils typically composed of a single protein [7]. The interaction between short (6–15 amino acids) and hydrophobic aggregation-prone regions (APRs) by β-strand interactions is the most prevalent structural mechanism driving protein aggregation [8, 9], and can give rise to both fibrillar and amorphous aggregates [10]. APRs are present in virtually all proteins composed of more than 150 amino acids and can be accurately predicted using computer algorithms such as WALTZ and TANGO [11]. A given proteome contains mostly unique APR sequences. However, a minority of redundant APRs (with an identical or homologous sequence) can be found in several or sometimes many different proteins [12, 13]. The exposed APRs tend to self-interact and form stable prefibrillar oligomers by rearranging themselves into a series of β-strands. These newly formed oligomers act as seeding intermediates and rapidly catalyse the growth of amyloids by the further recruitment of monomers that share the same or homologous APRs [14]. Thus, the addition of preformed aggregates (also called seeds) can induce the aggregation of identical proteins or homologous proteins [14–17]. This inspired the development of Pept-ins, which induce the aggregation of target proteins via APR interaction and subsequently the loss of function of target proteins. In Pept-ins, the aggregation seed concept is mimicked by combining two APRs on a single peptide sequence, which is reminiscent of the imperfect repeats that are observed in naturally occurring functional amyloids such as the yeast prion sup35 [18].

Pept-ins targeting the unique APR of target protein VEGFR2 of tumour cells and polymerase basic protein 2 in influenza are effective as anti-tumour and anti-virus strategies, respectively [19, 20]. The antimicrobial effect of Pept-ins is achieved via targeting redundant APRs in bacteria which induce the simultaneous aggregation of a large number of bacterial proteins, which are then sequestered in the inclusion bodies (IBs) at the polar regions. The formed IBs in Pept-in-treated bacteria can be visualised by the cross-section transmission electron microscopy (TEM) images and the structure of these IBs has been shown to be amyloid-like since they can be stained by amyloid-specific dye pFTAA and are enriched with β-sheet structure as shown by their Fourier Transform Infrared Spectroscopy (FTIR)-spectrum [12]. Many bacterial proteins which are associated with the APR of antimicrobial Pept-ins have been identified in the purified IBs by performing mass-spec proteomic analysis [12]. The trapping of many bacterial proteins in IBs eventually leads to the disruption of bacterial proteostasis and bacterial death [6]. The efficiency of antimicrobial Pept-ins has been extensively validated in gram-positive *S*. *aureus* or gram-negative *E*. *coli* and *A*. *baumannii* by targeting redundant APRs [6, 12]. Multitarget antibiotics have been shown to have a lower likelihood of inducing a high level of endogenous resistance [21, 22]. Indeed, we observed no Pept-in resistance development in wildtype *E*. *coli* and *S*. *aureus* [6, 12]. Even in hypermutable *E*. *coli* XL1-Red, which has a 5000-fold higher mutation rate, limited Pept-in resistance development was observed [23].

The main mode of action of these antimicrobial Pept-ins is achieved through inducing the aggregation of a large number of proteins in bacteria which eventually leads to IB formation at the polar regions [6, 12]. Many details of their mechanism of action remain to be investigated, which is crucial for successful antimicrobial Pept-in development, efficacy improvement and toxicity minimisation against mammalian cells. Further questions which remain to be explored include: (i) What determines Pept-in uptake efficiency and whether increased Pept-in uptake can improve Pept-in efficacy? (ii) Since Pept-ins exert the bactericidal effect via inducing protein aggregation, whether the ability of Pept-ins to induce aggregation in bacteria is positively related to antimicrobial potency? (iii) Does Pept-in antimicrobial activity depends on its secondary structure? (iv) We have previously shown that the resistant mechanism of both laboratory-derived and clinically isolated Pept-in-resistant strains is mainly achieved by reducing Pept-in uptake [23]. The question then becomes whether Pept-in structure modification can restore its accumulation and subsequently the antimicrobial activity against Pept-in resistant strains.

To answer the above-mentioned questions, we investigated the correlation between the structure of two previously discovered Pept-Ins and their antimicrobial potency. The impact of each structure modification on Pept-in antimicrobial potency, Pept-in uptake, aggregation properties *in vitro*, secondary structure and aggregation in bacteria was analysed. Results demonstrated that enhanced Pept-in antimicrobial potency due to structural modification is often associated with improved efficiency in accumulation and inducing protein aggregation in bacteria. These structural modifications can enhance the Pept-in therapeutic window by improving Pept-in antimicrobial potency and exhibiting no toxicity to mammalian cells.

## Results

### Arginine is essential for Pept-in mediated antimicrobial activity

We focused on previously identified Pept-in P2 (Table 1) [12] to investigate Pept-in structure and activity relationship due to its thoroughly investigated mode of action *in vitro* and efficacy in reducing bacterial load in a mouse model infected with *E*. *coli*. For some structural modifications, we also included Pept-in P33 (Table 1) to compare the effect of structural changes on antimicrobial potency with P2. To generate efficient seeds for inducing protein aggregation in bacteria, Pept-ins employ a tandem repeat design which includes two identical APRs flanked by three solubilising arginine residues (also called gatekeepers) and linked by a single proline (S1A Fig) [6, 12]. Given that functional amyloids in nature frequently contain multiple copies of the APR [7], we used two identical APRs in Pept-ins to improve the likelihood of seeding the aggregation of target proteins. The arginine gatekeepers are essential for the cellular uptake of antibacterial Pept-ins, in addition to their role in counteracting the high self-aggregation propensity of the core regions of the Pept-in.

**Table 1:** Amino acid sequence and MIC of Pept-in P2 and P33.

Peptide	Sequence	Peptide length (Amino acid)	BL21 MIC (μg/mL)
P2	RGLGLALVRRPRGLGLALVRR	21	12.5
P33	RLGIAVALRRPRLGIAVALRR	21	6.3

**Fig 1:**
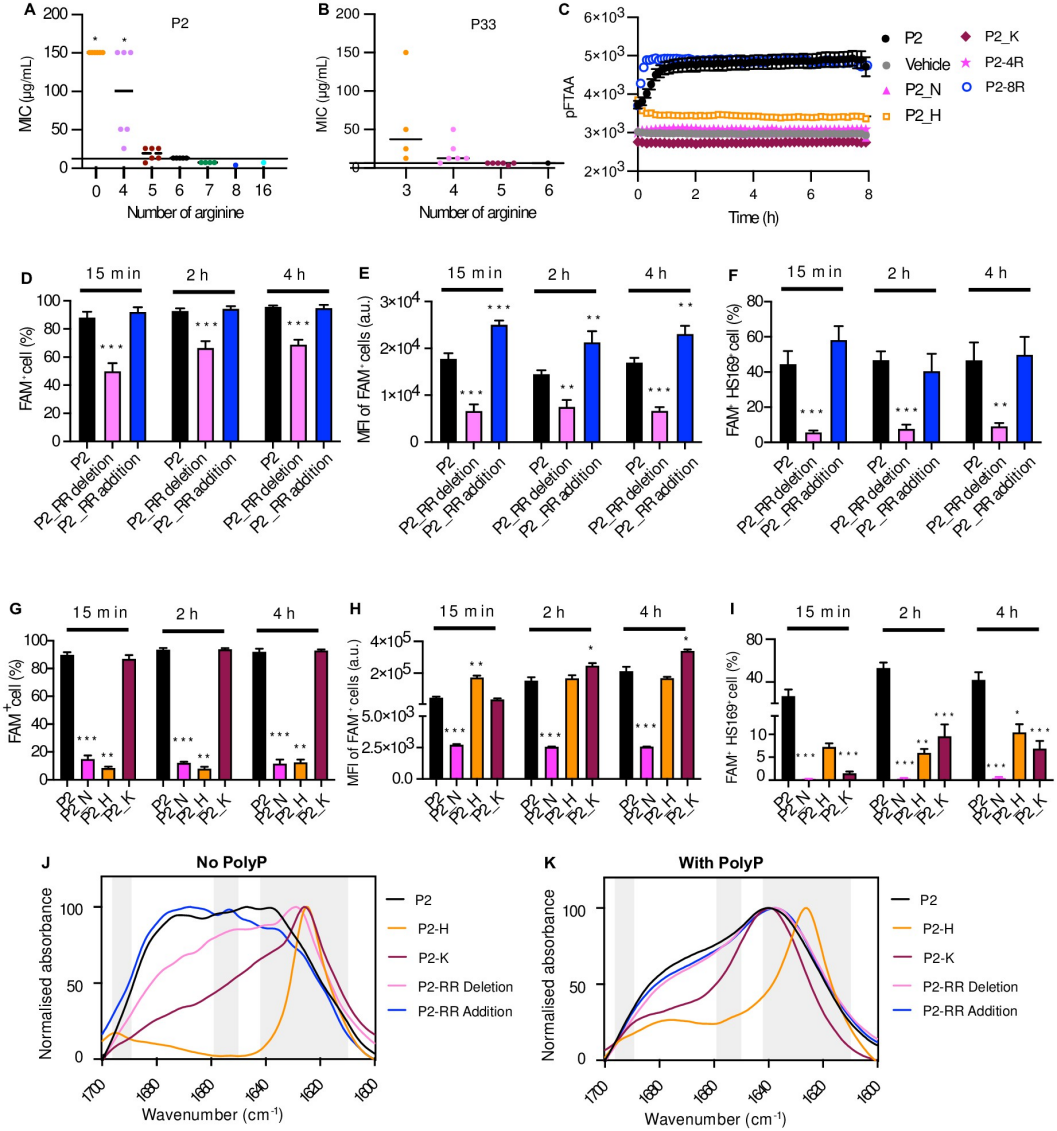
Arginine is essential for Pept-in mediated antimicrobial activity. **A-B:** MIC of P2 (**A**) and P33 (**B**) variants generated by modifying the amount of arginine against *E*. *coli* BL21. These two figures are associated with data from S1 and S2 Tables, respectively. Each dot represents the MIC of one Pept-in design. **C:** Time dependence of pFTAA (0.5 μM) fluorescence intensity of P2 derivatives (50 μM) in the presence of polyP (0.5 mM). (Error bars represent SEM, n = At least 3). **D-I:** Flow cytometry analysis of *E*. *coli* BL21 treated with FAM-labelled Pept-ins for different time points from three independent experiments. Samples from D-E were acquired using BD Fortessa X-20, whereas samples from G-I were acquired using Gallios Flow Cytometry. The percentage of FAM positive cells (**D**), FAM MFI of FAM positive cell population (**E**), and the percentage of FAM and HS169 positive cells (**F**) when treated with P2 or its derivatives with a reduced or an increased amount of arginine residues at the concentration of FAM-P2 RR addition MIC. The percentage of FAM positive cells (**G**), FAM MFI of FAM positive cell population (**H**), and the percentage of FAM and HS169 positive cells (**I**) when treated with P2 or its derivatives with replaced gatekeepers at the concentration of FAM-P2 MIC (25 μg/mL). Error bars represent SEM for (D-I), n = 9 in D, E and F, n = at least 3 in G, H and I. **J-K:** FTIR spectrum of P2 variants with altered gatekeepers in PBS (6% DMSO), without (**J**) or with (**K**) the presence of polyP. The absorbance is normalised between all samples and the spectrum is scaled to the amide I region between 1600–1700 cm^−1^. Peaks within the left (1689–1696 cm^−1^) and right (1610–1642 cm^−1^) grey bar are assigned to β-sheet, while peaks within the grey bar in the middle (1651–1659 cm^−1^) is assigned to α-helix. The FTIR spectrums are representative of three independent experiments. For **A** and **B**, one-sample Wilcoxon signed-rank test was used to compare the MIC median of each Pept-in group to P2 MIC (12.5 μg/mL) or P33 MIC (6.25 μg/mL). For **D-I**, a two-tailed Student t-test was performed for calculating statistical significance between the mean of P2 variant and P2. Asterisks indicating the level of the p value centred over the error bar mean: *p < 0.05, **p < 0.01, ***p < 0.001, and ****p < 0.0001.

Positively charged amino acids (arginine: R, lysine: K, histidine: H) of antimicrobial peptides (AMPs) play a critical role in their antibacterial activity by promoting peptide uptake or membrane permeabilization [24, 25]. Antimicrobial Pept-ins such as P2 and P33 typically contain six arginine residues, with three arginines flanking the side of each APR (Table 1). To further assess the role of these arginine residues in P2 and P33 mediated antimicrobial activity, we first modified the number of arginines and determined the corresponding impact on antimicrobial activity. Reducing the number of arginine residues from six, by either directly deleting or replacing arginine with an uncharged amino acid, leading to higher minimal inhibitory concentrations (MICs) for both P2 and P33 (Fig 1A and 1B, S1 and S2 Tables). An increase in the number from six to seven or eight resulted in (slightly) lower P2 MICs, although no further improvement was observed when it increased to sixteen (Fig 1A, S1 Table). Interestingly, the replacement of arginine for P2 with positively charged lysine or histidine led to a loss of antimicrobial activity (MIC > 100 μg/mL (S1 Table). Thus, arginine is essential for the antimicrobial activity of P2 and P33, and the amount of arginine positively correlates with Pept-in antimicrobial potency, albeit this positive correlation is limited to eight arginine residues.

Next, we investigated whether arginine distribution plays a role in the observed antimicrobial activity. For P2, changes in arginine distribution led to MIC variation between 25 μg/mL and > 100.0 μg/mL when the net charge is +4 (Fig 1A, S1 Table). However, the MIC is less affected for P2 variants with a higher net charge (+5, +6, or +7) (Fig 1A, S1 Table). A similar phenomenon was observed for P33 variants as well (Fig 1B, S2 Table), arginine distribution modification resulted in varied MIC values between 12.5 and > 100 when the net charge is +3. However, 83% of P33 variants showed unaffected MIC values when the net charge is +5 (Fig 1B, S2 Table). These results indicate that charge distribution plays a less important role for Pept-in antimicrobial potency when the net charge is high, but it can be a determining factor for Pept-in antimicrobial potency when the net charge is low.

### Arginine improves Pept-in efficiency to accumulate and to induce protein aggregation in bacteria

To understand how structural modifications affect Pept-in antimicrobial activity, Pept-in variants with both enhanced and reduced antimicrobial activity were included in this study to investigate the impact of structural modification on a range of activities, including Pept-in accumulation and Pept-in-induced aggregation in bacteria. Pept-in accumulation efficiency in bacteria was determined by treating bacteria with 5-carboxyfluorescein (FAM)-labelled Pept-in at the concentration of FAM-P2 MIC and then measuring the percentage of FAM^+^ cells. Additionally, we determined the median fluorescence intensity (MFI) of FAM within FAM^+^ cells to quantify the accumulated peptide in bacteria. The bacterial population with Pept-in induced protein aggregation events was identified by staining bacteria with the amyloid-specific dye HS-169 [26] and then determining the percentage of FAM^+^HS-169^+^ cells. Deleting two arginine residues (P2_RR_Deletion, MIC > 100 μg/mL) led to lower efficiency in accumulating in bacteria as demonstrated by a lower percentage of FAM^+^ cells and a lower MFI of FAM^+^ cells compared to FAM-P2 treated cells (Fig 1D and 1E, S1B Fig). Although about 70% of bacteria were FAM^+^ after 4 h treatment of FAM_P2_RR_Deletion (Fig 1D), only about 10% showed protein aggregation events (represented by FAM^+^HS169^+^ cells) (Fig 1F, S1B Fig), which could be caused by the lower amount of accumulated peptide compared to FAM_P2. Adding two arginine residues (P2_RR_Addition, MIC 3.1 μg/mL) led to a higher MFI of FAM within FAM^+^ cells (Fig 1E, S1B Fig), while it did not change the percentage of FAM^+^ cells and FAM^+^HS169^+^ cells (Fig 1D and 1F, S1B Fig). These results demonstrated that the number of arginine residues positively correlates with its antimicrobial potency of P2 by affecting Pept-in accumulation efficiency and/or the subsequent ability to induce protein aggregation in bacteria.

Next, we investigated whether replacing arginine to uncharged asparagine (N) or other positively charged amino acids (Histidine: H, lysine: K) also disrupted Pept-in accumulation or pept-in induced aggregation in bacteria.

Both P2_N and P2_H almost completely obliterated Pept-in accumulation since only about 10% of bacteria were FAM^+^ even after 4 h treatment (Fig 1G, S1C Fig). Subsequently, only about 1% and 10% of FAM^+^HS169^+^ bacteria were observed after 4 h treatment of P2_N and P2_H, respectively (Fig 1I). It is worth noting that although replacement of arginine to lysine (P2_K) caused a higher amount of Pept-in accumulation in all bacterial cells (Fig 1H), only 6% of bacteria were FAM^+^HS169^+^ after 4 h of treatment (Fig 1I, S1C Fig), showing that lysine mediates uptake but prevents efficient aggregation. It remains to be investigated why histidine cannot promote Pept-in uptake and why lysine as gatekeepers leads to protein aggregation in fewer bacteria, but these results collectively show that both intracellular accumulation and the subsequent protein aggregation events are necessary for the antimicrobial activity of P2.

### Arginine is an optimal gatekeeper in balancing Pept-in aggregation propensity with peptide solubility

Arginine as a gatekeeper in Pept-ins can prevent massive Pept-in aggregation and thereby increases their solubility. Therefore, we hypothesised that altered gatekeepers could also affect Pept-in *in vitro* aggregation properties and thereby having an impact on their antimicrobial potency. To investigate Pept-in *in vitro* aggregation behaviour, we used the amyloid-specific dye pentameric formyl thiophene acetic acid (pFTAA), which identifies several intermediate species occurring during protein aggregation, including the soluble pre-fibrillar and oligomeric species, amorphous aggregates, as well as mature fibrillar structures [27]. We further analysed Pept-in secondary structure using FTIR (Table 2) [28–30], with or without the presence of polyphosphate (polyP). PolyP is abundant in bacteria and human cells [31, 32] and can accelerate protein aggregation.

**Table 2:** FTIR secondary structure assignments.

Wavenumber (cm^-1^)	Structure assignment
1610–1642	β-sheet
1643–1650	Random coil
1651–1659	α-helix
1660–1688	β-turn
1689–1696	β-sheet

P2 in PBS (6% DMSO) did not display pFTAA-positive aggregation over time as shown by the straight line of pFTAA (S2A Fig). Correspondingly, the FTIR spectrum of P2 showed a broad absorbance band between 1635 cm^-1^ and 1672 cm^-1^, indicating the presence of different secondary structures including β-sheets, α-helix, random coils, and β-turns (Table 2, Fig 1J). In contrast, P2 aggregated rapidly in the presence of polyP as demonstrated by a sharp increase in pFTAA fluorescence intensity (Fig 1C). At the end of the kinetic experiment (8 h), TEM images revealed the formation of large amorphous aggregates with a length up to 4 μm (S2B Fig). The FTIR spectrum showed that these amorphous aggregates have a predominant peak around 1635 cm^-1^ and a small shoulder around 1686 cm^-1^, indicating the shift to a β-sheet enriched structure (Fig 1K). The requirement of polyP to promote P2 aggregation in PBS also indicates the strong gatekeeping effect of arginine.

In the absence of polyP, no increase in pFTAA fluorescence intensity was observed for all P2 variants with altered gatekeepers (S2A Fig). However, we observed the formation of precipitates for P2_H and P2_K, indicating the formation of insoluble aggregates despite no increase in pFTAA intensity. In agreement with this observation, P2_H and P2_K showed a sharp peak around 1625 cm^-1^ and low absorbance in the range between 1641 cm^-1^ and 1672 cm^-1^ (Fig 1J), indicating the formation of predominantly β-sheets. While the FTIR spectrum of P2-RR addition displayed a broad absorbance band similar to P2, P2-RR deletion had a strong maximum around 1627 cm^-1^ and weak peaks in the range between 1641 cm^-1^ and 1675 cm^-1^ (Fig 1J), suggesting that deleting two arginine residues led to increased formation of β-sheets. These results collectively suggest that the decreased gatekeeping effect, either due to replacing arginine with histidine or lysine, or decreasing the number of arginines, could result in lower efficiency in preventing Pept-in aggregation and keeping Pept-in in a solubilised state, thereby contributing to the loss of antimicrobial activity.

In the presence of polyP, we observed an altered *in vitro* aggregation behaviour for the ones with decreased antimicrobial potency, such as no increase in pFTAA fluorescence intensity (Fig 1C), the formation of small aggregates (P2_N: around 250 nm, P2_RR_Deletion: around 500 nm), or with a distinct aggregate morphology (P2_H: Fibrils, P2_K: Both fibrillar and amorphous aggregates) (S2B Fig). Corresponding to the distinct aggregate morphology of P2_H and P2_K, P2-H had a FTIR maximum at 1625 cm^-1^ instead of 1635 cm^-1^, while P2_K had lower absorbance in the range between 1641 cm^-1^ and 1675 cm^-1^ than P2 (Fig 1K), further confirming that Pept-in secondary structure can be altered due to gatekeeper replacement. The Pept-in with increased antimicrobial potency (P2_RR addition) showed a comparable pFTAA kinetic (Fig 1C) and FTIR spectrum like P2 (Fig 1K), as well as formed similar amorphous aggregates as P2 (S2B Fig). Therefore, arginine is an optimal gatekeeper in balancing Pept-in aggregation propensity, by keeping Pept-ins in small and soluble states in solution and allowing Pept-ins to aggregate in the presence of aggregation-promoting molecules which represent the bacterial environment. This balanced Pept-in aggregation propensity with peptide solubility seems to be pivotal for its antimicrobial function in bacteria.

### Increased aggregation propensity enhances Pept-in antimicrobial potency by promoting Pept-in accumulation and protein aggregation events in bacteria

Since we observed that inducing protein aggregation in bacteria is critical for Pept-in mediated antimicrobial activity, we then investigated whether there is a direct relationship between Pept-in aggregation propensity and its antimicrobial potency. The aggregation propensity of a peptide sequence can be obtained using TANGO [11], which predicts its β-aggregation propensity in a sequence-specific manner based on the assumption that a highly aggregation-prone peptide has a high tendency in forming β-sheets. TANGO returns a score for each amino acid of a given sequence in the range of 1–100, and a sequence with five consecutive amino acids with a TANGO score above five is predicted to be aggregation-prone [11]. Therefore, to compare the aggregation propensity between a Pept-in and its variants, we compared the sum of the TANGO score for the amino acids of the APR.

**Fig 2:**
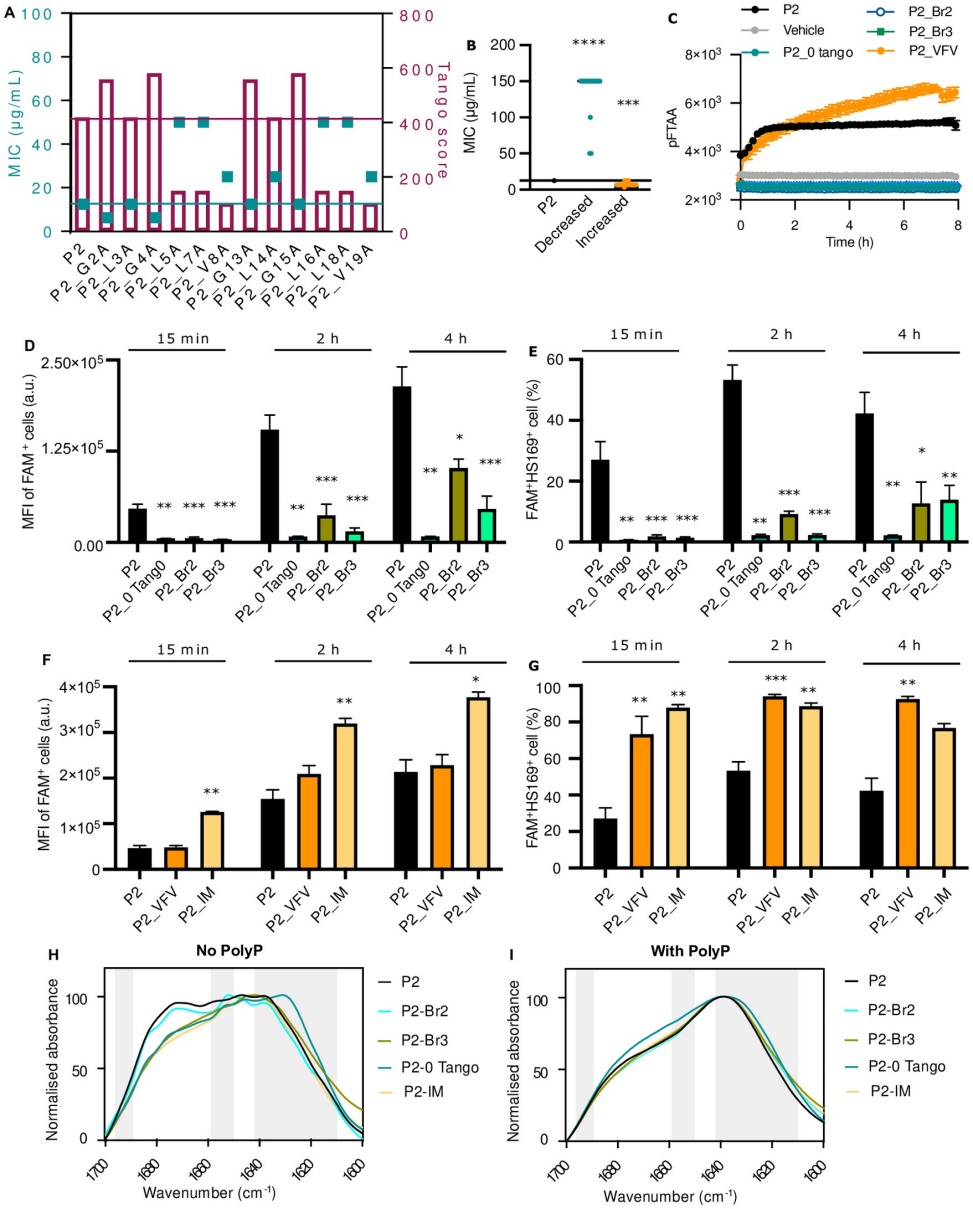
Increased aggregation propensity enhances Pept-in antimicrobial potency by promoting Pept-in accumulation and IB formation. **A:** MIC (left Y-axis) against *E*. *coli* BL21 and Tango score (right Y-axis) of P2 derivatives generated by alanine scanning of the APR region. This figure is associated with data from S3 Table. The red bars represent the APR TANGO score, and the red line is the baseline of the P2 APR Tango score. The blue squares represent MIC values, and the blue line is the baseline of P2 MIC. **B**: MIC of P2 variants generated by aggregation propensity modification against *E*. *coli* BL21. This figure is associated with data from S5 Table. Each dot represents the MIC of one Pept-in design. **C-G:** P2 variants with decreased aggregation propensity: P2_0 Tango, P2_Br2, P2_Br_3; with increased aggregation propensity: P2_VFV, P2_IM. **C:** Time dependence of pFTAA (0.5 μM) fluorescence intensity of P2 derivatives (50 μM) in the presence of polyP (0.5 mM) (n = at least 6). **D-G:** Flow cytometry analysis of *E*. *coli* BL21 treated with FAM-labelled Pept-ins for different time points at FAM-P2-MIC from three independent experiments. Samples were acquired using Gallios Flow Cytometry. MFI of FAM^+^ cells (**D**), and the percentage^+^ of FAM and HS169^+^ cells (**E**) when treated with P2 and its derivatives with decreased antimicrobial potency. MFI of FAM^+^ cells (**F**), and the percentage^+^ of FAM and HS169^+^ cells (**G**) when treated with P2 and its derivatives with increased antimicrobial potency. Error bars represent SEM. **H-I:** FTIR spectrum of P2 variants with altered aggregation propensity in PBS (6% DMSO), without (**H**) or with (**I**) the presence of polyP. The absorbance is normalised between all samples and the spectrum is scaled to the amide I region between 1600–1700 cm^−1^. Peaks within the left (1689–1696 cm^−1^) and right (1610–1642 cm^−1^) grey bar are assigned to β-sheet, while peaks within the grey bar in the middle (1651–1659 cm^−1^) is assigned to α-helix. The FTIR spectrums are representative of three independent experiments. For **B**, one-sample Wilcoxon signed-rank test was used to compare the MIC median of each Pept-in group to P2 MIC (12.5 μg/mL). For **D-G**, a two-tailed Student t-test was performed for calculating statistical significance between the mean of P2 variant and P2 (n = at least 6). Asterisks indicating the level of the p value centred over the error bar mean: *p < 0.05, **p < 0.01, ***p < 0.001, and ****p < 0.0001.

The TANGO score of P2 APR is 419.9, alanine scanning of P2 APR generated six P2 derivatives with reduced TANGO scores in the range of 104–150, indicating a decreased aggregation propensity [11], and all six derivatives displayed higher MICs against *E*. *coli*. (Fig 2A, S3 Table). While for P33, eight derivatives with reduced APR TANGO scores in the range of 270–450 were generated by alanine scanning, here only 37.5% of P33 derivatives showed higher MICs (S3A Fig, S3 Table). The reason why the effect of a lower Tango score had less effect on P33 is likely due to the fact that although the Tango score is lower than the original P33, however, it is still high enough for the variant to be effective. These data suggest that Pept-in aggregation propensity is negatively correlated with its MIC in a non-linear manner and affects Pept-in antimicrobial activity the strongest in the lower range. Systematic truncation of P2-WG from the C-terminus led to increased MIC, and this increase was positively related to the number of deleted amino acids (S4 Table), further confirming that Pept-in mediated antimicrobial activity is closely related to the number of arginine residues, APR Tango score and the tandem repeat design.

To further explore this observation, we generated P2 derivatives with decreased aggregation propensity by APR modification which results in 0 TANGO score of P2 APR, aggregation-sweeping entropic bristle addition [33], or glycosylation [34]. Entropic bristles are intrinsically disordered and highly charged sequences, the addition of which to an aggregation-prone protein is able to prevent protein aggregation by excluding large particles due to the random movement of the entropic bristles [33]. While 2 out of these 18 derivatives showed a MIC increase from 12.5 μg/mL to 50 μg/mL, the rest all displayed a MIC at > 100 μg/mL (Fig 2B, S5 Table). In contrast, 11 out 14 P2 derivatives with an increased APR Tango score showed a MIC decrease from 12.5 μg/mL to 6.3 or 3.1 μg/mL (Fig 2B, S5 Table); while 3 variants, for example, P2-VFV with the highest TANGO score, showed the same antimicrobial activity as P2 (S5 Table). A non-linear negative correlation was found between the P2 APR TANGO score and P2 MIC with a correlation coefficient of -0.76 (S3B Fig). These results collectively demonstrated that Pept-in aggregation propensity needs to be carefully assessed in order to optimise its antibacterial activity, since up to a certain threshold, increasing aggregation propensity promotes Pept-in antimicrobial potency.

To further investigate why modifying Pept-in APR aggregation propensity affects its antimicrobial potency, we determined the accumulation as well as protein aggregation in bacteria for the following P2 variants: with lower antimicrobial potency (P2_0 Tango score, P2_Br2, P2_Br3), unaffected antimicrobial potency (P2_VFV), and higher antimicrobial potency (P2_IM). Corresponding with the observation that variants with less antibacterial activity exhibited lower efficiency in accumulating in bacteria, P2_0 Tango score demonstrated a lower MFI of the FAM^+^ cells (Fig 2D), while P2 with bristle addition (P2_Br2 and P2_Br3) showed both a lower percentage of FAM^+^ bacterial cells as well as a lower MFI of the FAM^+^ cells (Fig 2D, S3C Fig). In contrast, P2_IM treated bacteria were all FAM^+^ after 15 min treatment (S3C Fig) and showed a higher MFI of the FAM^+^ cells than FAM_P2 treated bacteria (Fig 2F). Correspondingly, P2_0 Tango score, P2_Br2, and P2_Br3 induced protein aggregation in fewer bacteria as shown by the lower percentage of FAM^+^HS-169^+^ cells (Fig 2E), whereas P2_IM treated bacteria showed a higher percentage of FAM^+^HS-169^+^ cells (Fig 2G). Therefore, data here further confirmed that Pept-in accumulation in bacteria and/or Pept-in induced aggregation events are positively correlated with its antimicrobial potency. However, it should be noted that P2_VFV with the highest Tango score also induced protein aggregation in a higher percentage of cells (FAM^+^HS-169^+^ cells) (Fig 2G), even though it has a similar MIC as P2 against *E*. *coli*.

Regarding the *in vitro* aggregation behaviour, no increase in pFTAA fluorescence intensity (S3D Fig) and a wide absorbance band (between 1628 cm^-1^ and 1680 cm^-1^) (Fig 2H) were observed for P2 and P2-Br2, P2-Br3, P2-0-Tango, and P2-IM in the absence of polyP. However, the derivative with the highest Tango score (P2_VFV) showed a sharp increase in pFTAA intensity (S3D Fig), confirming that P2_VFV indeed has the highest aggregation propensity as predicted by TANGO. In the presence of polyP, P2 derivatives with decreased antimicrobial potency did not show any increase in pFTAA fluorescence intensity (Fig 2C) and only small (about 200 nm) aggregates were occasionally observed (S3E Fig), whereas P2_VFV showed similar aggregation behaviour as P2 (S2E Fig). The similar FTIR spectrum of P2 variants with bristle addition (P2-Br2 and P2-Br3) and decreased aggregation propensity (P2-0-Tango) suggests that they can form small aggregates with a similar β-sheet structure as P2 (Fig 2I), but not able to grow into large aggregates (S3E Fig). These results further confirm that the ability of Pept-ins to aggregate into large aggregates in the presence of polyP is important for its antimicrobial function in bacteria.

### Optimizing the aggregate core structure enhances Pept-in antimicrobial potency

**Fig 3:**
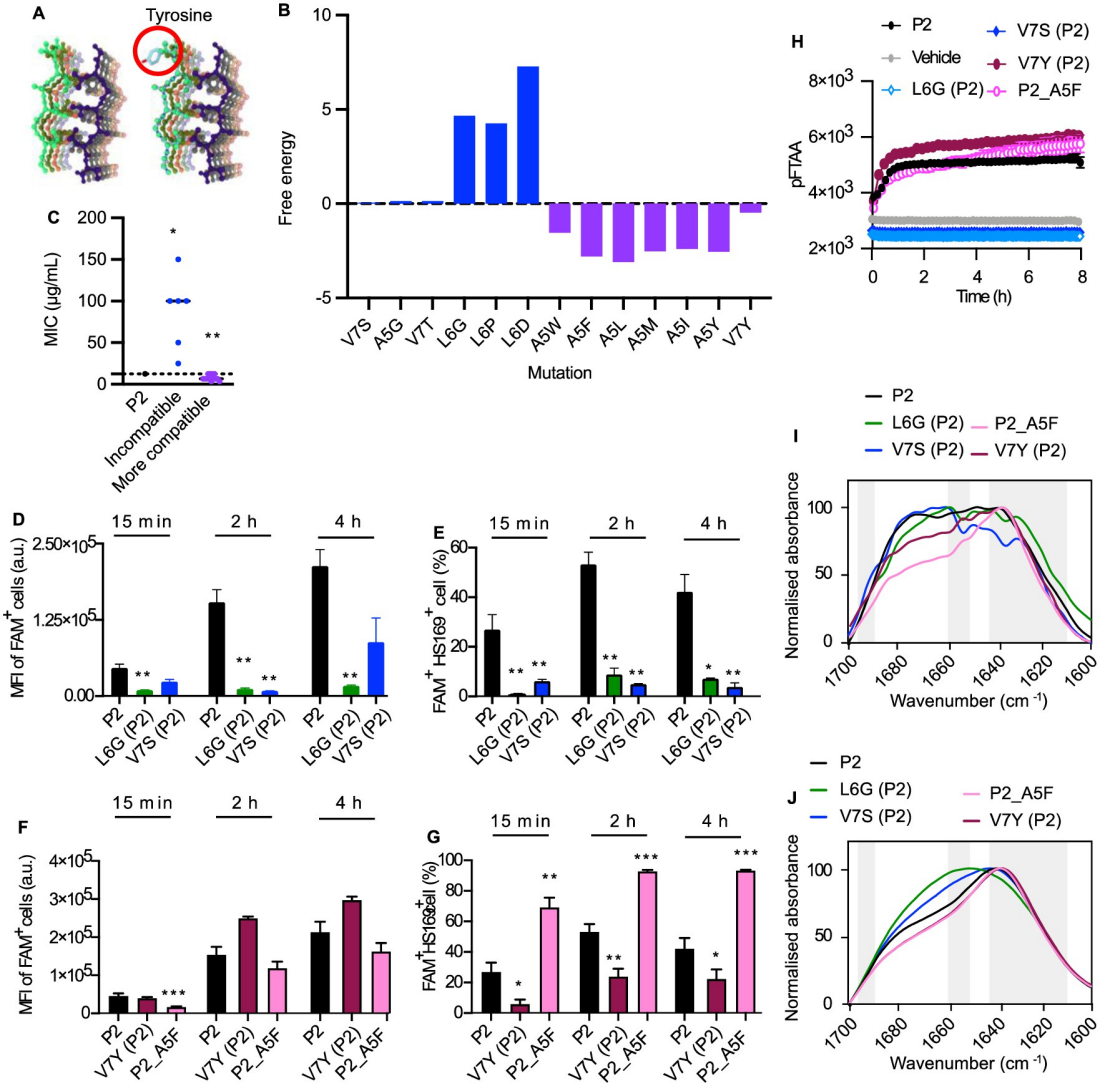
Optimizing the aggregate core structure enhances Pept-in antimicrobial potency. **A:** The most likely structure predicted by Cordax for the amyloid core formed between P2 (**left**) and the APR of target proteins. The impact of introducing a mutant in the APR (e.g., V7Y) (**right**) can then be evaluated using the FoldX force field. **B:** Free energy of the APR structure calculated by FoldX after introducing a single mutation in the APR region. Blue bars represent mutations that are incompatible with the cross-beta core structure, while purple bars represent the ones that are more compatible with the cross-beta core structure. **C**: MIC of P2 variants generated by modifying the aggregate core structure against *E*. *coli* BL21. This figure is associated with data from S6 Table. Each dot represents the MIC of one Pept-in design. **D-H:** P2 variants which are incompatible with the cross-beta core structure: L6G (P2); V7S (P2); P2 variants which are more compatible with the cross-beta core structure more compatible: V7Y (P2), P2_A5F. **D-G:** Flow cytometry analysis of *E*. *coli* BL21 treated with FAM-labelled Pept-ins for different time points at FAM-P2-MIC from three independent experiments. Samples were acquired using Gallios Flow Cytometry. MFI of FAM^+^ cells (**D**), and the percentage^+^ of FAM and HS169^+^ cells (**E**) when treated with P2 and its derivatives with decreased antimicrobial potency. MFI of FAM^+^ cells (**F**), and the percentage^+^ of FAM and HS169^+^ cells (**G**) when treated with P2 and its derivatives with increased antimicrobial potency. Error bars represent SEM. **H:** Time dependence of pFTAA (0.5 μM) fluorescence intensity of P2 derivatives (50 μM) in the presence of PolyP (0.5 mM). (n = at least 6). **I-J:** FTIR spectrum of P2 variants with altered compatibility to the aggregate core structure in PBS (6% DMSO), without (**I**) or with (**J**) the presence of PolyP. The absorbance is normalised between all samples and the spectrum is scaled to the amide I region between 1600–1700 cm^−1^. Peaks within the left (1689–1696 cm^−1^) and right (1610–1642 cm^−1^) grey bar are assigned to β-sheet, while peaks within the grey bar in the middle (1651–1659 cm^−1^) is assigned to α-helix. The FTIR spectrums are representative of three independent experiments. For **C**, one-sample Wilcoxon signed-rank test was used to compare the MIC median of each Pept-in group to P2 MIC (12.5 μg/mL). For **D-G**, a two-tailed Student t-test was performed for calculating statistical significance between the mean of P2 variant and P2 (n = at least 6). Asterisks indicating the level of the p value centred over the error bar mean: *p < 0.05, **p < 0.01, ***p < 0.001, and ****p < 0.0001.

Given the importance of the aggregation propensity of P2, we decided to try a structure-based approach to design further variants. To this end, we first modelled the core of the APR portion of P2 in the cross-beta state using Cordax, a machine learning algorithm that predicts the most likely cross-beta topology of a given APR sequence [35] (Fig 3A, Left). Next, we used the FoldX force field [36] to model the effect of introducing single side chain variations on the free energy of the APR structure (Fig 3A, right) [37]. A negative value in this energy suggests a higher tendency to form the cross-beta structure than the wild type, while a positive value suggests the opposite (Fig 3B). This allowed identifying a number of derivatives that are predicted to be more compatible or incompatible with the cross-beta core structure (Fig 3B). All P2 variants predicted in this manner to be incompatible with the cross-beta structure exhibited decreased antimicrobial potency against *E*. *coli* with a MIC increase (Fig 3C, S6 Table). On the contrary, 69% of P2 variants with a predicted more favourable energy in the cross-beta structure showed increased antimicrobial potency, while 31% exhibited unaffected antimicrobial activity (Fig 3C, S6 Table). Therefore, it seems that these structure-based designs can be used to enhance Pept-in antimicrobial potency.

We next investigated the correlation between Pept-in antimicrobial potency and Pept-in accumulation as well as Pept-in-induced protein aggregation in bacteria. For this purpose, we selected P2 variants with lower antimicrobial potency (L6G (P2), V7S (P2)), and higher antimicrobial potency (P2_A5F and V7Y(P2). All bacteria showed accumulation of P2 and P2 variants after only 15 min treatment (S2C Fig), independently of their antimicrobial activity. However, P2 variants with a lower antimicrobial potency showed a reduced ability to accumulate in bacteria compared to P2, as demonstrated by a significantly lower MFI of FAM^+^ bacterial cells (Fig 3D). Correspondingly, these derivatives induced protein aggregation in fewer bacteria as shown by the lower percentage of FAM^+^HS-169^+^ cells (Fig 3E). Interestingly, although both P2_A5F and V7Y (P2) exhibited a similar kinetic in accumulating (similar MFI as P2, Fig 3F), P2_A5F induced protein aggregation in a higher percentage of bacteria compared to P2 (Fig 3G, S4B Fig), whereas V7Y (P2) induced protein aggregation in a lower percentage of bacteria (Fig 3G). These results correspond to the observation that both Pept-in accumulation in bacteria and Pept-in induced aggregation events are critical for exerting its antimicrobial activity.

Next, we checked whether Pept-in antimicrobial activity is correlated with its *in vitro* aggregation property as observed for P2 variants with altered gatekeepers. Despite no pFTAA increase was observed for P2 and all variants in the absence of polyP (Fig 2D), we observed a difference in their FTIR spectrums. While L6G (P2) and V7S (P2) showed a broad absorbance band between 1635 cm^-1^ and 1672 cm^-1^ as P2, P2_A5F and V7Y (P2) displayed a predominant peak around 1635 cm^-1^ (Fig 3I). This suggests that a more compatible structure to the amyloid core can promote Pept-in β-sheet structure formation. In the presence of polyP, no increase in pFTAA fluorescence intensity (Fig 2H) and formation of small aggregates (around 500 nm) (S2E Fig) were observed for P2 variants with lower antimicrobial potency (L6G (P2), V7S (P2)). In contrast, the ones with a higher antimicrobial activity (P2_A5F and V7Y (P2)) showed a similar pFTAA kinetic as P2 (Fig 3H) and formed large amorphous aggregates (around 12 μm) (S2E Fig). Correspondingly, P2, P2_A5F and V7Y (P2) had a predominant peak around 1635 cm^-1^, whereas L6G (P2) and V7S (P2) showed a wide absorption band between 1635 cm^-1^ and 1670 cm^-1^ (Fig 3J), confirming the more abundant presence of β-sheet structure for P2_A5F and V7Y (P2). These results further confirm the observation that the ability of Pept-ins to form β-sheet enriched aggregates in the presence of polyP is important for its antimicrobial function in bacteria.

### β-turn-promoting motifs and disulphide bond formation improve antimicrobial potency

**Fig 4:**
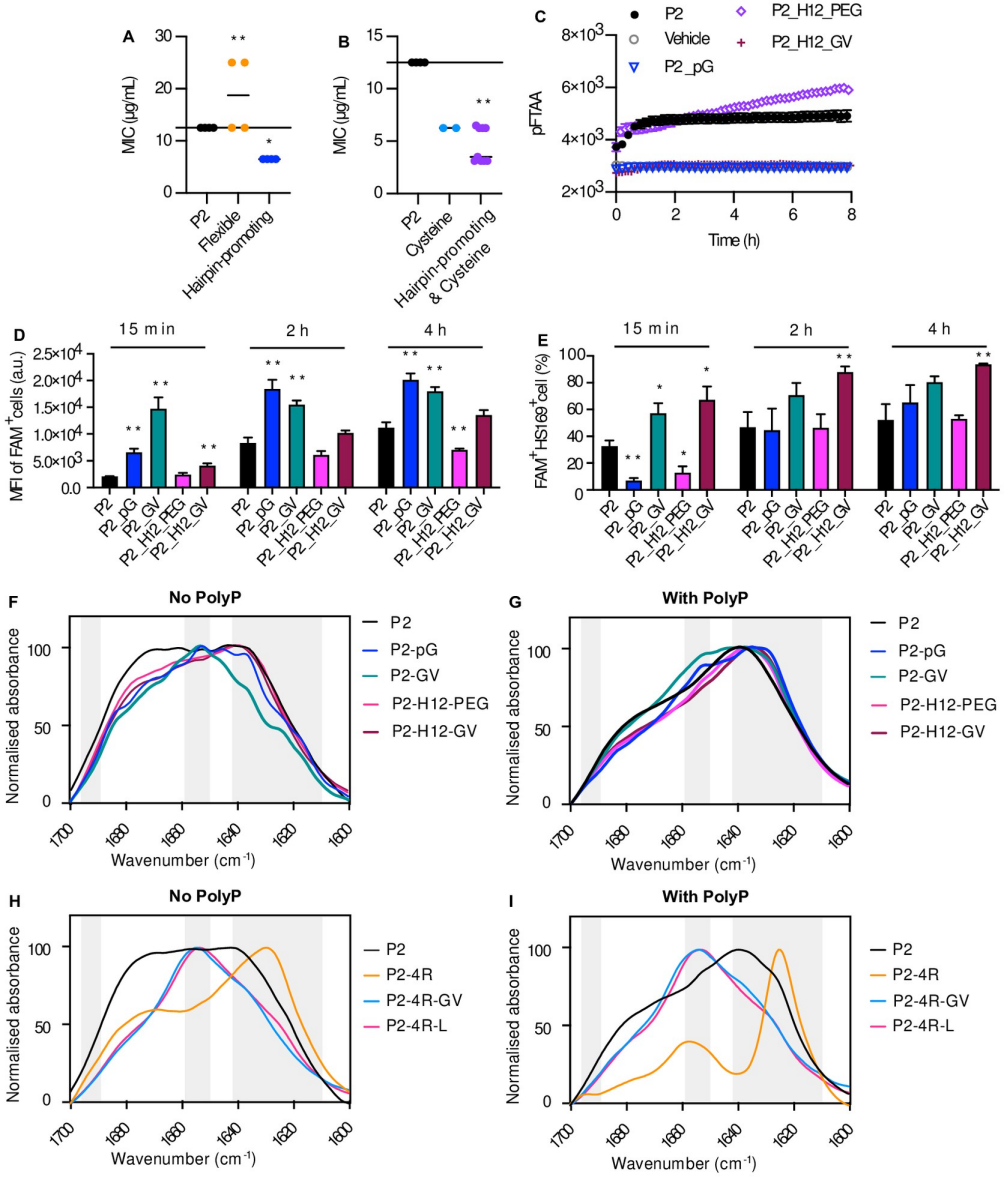
Beta-hairpin promoting motifs can improve antimicrobial potency. **A-B:** MIC of P2 variants generated by linker modification (**A**) or cysteine addition (**B**) against *E*. *coli* BL21. These two figures are associated with data from S7 and S8 Tables, respectively. Each dot represents the MIC of one Pept-in design. **C:** Time dependence of pFTAA (0.5 μM) fluorescence intensity of P2 derivatives (50 μM) in the presence of polyP (0.5 mM). (n = At least 3). **D-E:** Flow cytometry analysis of *E*. *coli* BL21 treated with FAM-labelled Pept-ins for different time points at FAM-P2-MIC from three independent experiments. Samples were acquired using BD Fortessa X-20. MFI of FAM positive cell population (**D**), and the percentage of FAM and HS169 positive cells (**E**) when treated with P2 and its derivatives with increased antimicrobial potency at the concentration of FAM-P2 MIC. Error bars represent SEM (n = 6). **F-I:** FTIR spectrum of P2 variants with altered linkers in PBS (6% DMSO), without (**F**, **H**) or with (**G**, **I**) the presence of PolyP. The absorbance is normalised between all samples and the spectrum is scaled to the amide I region between 1600–1700 cm^−1^. Peaks within the left (1689–1696 cm^−1^) and right (1610–1642 cm^−1^) grey bar are assigned to β-sheet, while peaks within the grey bar in the middle (1651–1659 cm^−1^) is assigned to α-helix. The FTIR spectrums are representative of three independent experiments. For **A-B**, one-sample Wilcoxon signed-rank test was used to compare the MIC median of each Pept-in group to P2 MIC (12.5 μg/mL). For **D-E**, a two-tailed Student t-test was performed for calculating statistical significance between the mean of P2 variant and P2. Asterisks indicating the level of the p value centred over the error bar mean: *p < 0.05, **p < 0.01, ***p < 0.001, and ****p < 0.0001.

Proline is a rigid residue that could be beneficial for Pept-in antimicrobial activity since it enables an extended structure between the two APRs of Pept-ins and thereby improving Pept-in solubility. However, replacement of proline by alanine or flexible linkers (with a length of 2–3 amino acids) [38] did not affect P2 MIC (Fig 4A, S7 Table), suggesting that Pept-ins exert their antimicrobial activity with a comparable efficacy when modifying the conformational rigidity and flexibility. Although, Pept-in antimicrobial potency reduced when the length of the flexible linker increased to 4 amino acids (S7 Table).

Next, we tested the impact of β-turn-promoting linkers on P2 antimicrobial potency, such as the reverse turn motif pG [39, 40], which promotes β-hairpin formation and allows a more defined structure [41] by introducing a reverse turn [42]. The cyclic structure formed between the side chain and the N-H of proline (P) offers a turn and a structure rigidity, making proline ideal for a β-turn [43]. Additionally, the short side chain of glycine (G) enables peptides to make a sharp turn as well. Applying various β-turn-promoting linkers all improved Pept-in potency by 2-fold (Fig 4A, S7 Table), indicating a consistent effect of these β-turn-promoting linkers. Additionally, introducing a disulphide bond formed by cysteine oxidisation, especially when combined with β-turn-promoting linkers, can improve P2 antimicrobial potency, with some linkers having a better effect than the others (Fig 4B, S8 Table). Therefore, these β-turn promoting motifs and disulphide bond formation appear to be beneficial for Pept-in antimicrobial activity.

While P2-H12-PEG demonstrated a comparable kinetic profile as P2 in accumulating and inducing protein aggregation in bacteria when treated at the concentration of FAM-P2 MIC, the other variants all showed a faster kinetic profile than P2 (Fig 4D and 4E, S5A Fig). This corresponds to the observation that increased Pept-in accumulation and/or Pept-in-induced protein aggregation events in bacteria are associated with enhanced Pept-in antimicrobial potency. Similar to P2, the variants with altered linkers did not show an increase in pFTAA fluorescence intensity in the absence of polyP (S5B Fig). P2_GV, P2_H12_PEG and P2_H12_GV showed a more predominant peak near 1635 cm^-1^ than P2 (Fig 4F), indicating a higher tendency in forming β-sheets. Interestingly, P2-GV formed mostly α-helix structures as demonstrated by the sharp peak around 1652 cm^-1^ (Fig 4F). In the presence of polyP, P2 variants with β-turn promoting motifs formed large aggregates (2–4 μm) containing a mixture of amorphous and fibrillar species (S5C Fig), exhibiting either a similar pFTAA kinetics as P2 or no pFTAA increase (Fig 4C). Despite the morphological difference of formed aggregates, these variants showed a similar FTIR spectrum as P2 with a sharp peak near 1635 cm^-1^, confirming the formation of predominantly β-sheet structures (Fig 4G). Therefore, the FTIR data could not confirm that alteration of the linkers or the formation of disulphide bond had a significant impact (p > 0.05 for each wavenumber when performing a paired nonparametric multiple-t test) on Pept-in secondary structure or β-turn confirmations upon aggregating.

### Structural modifications for improving Pept-in antimicrobial potency do not show synergy

Pept-in structure modification showed that an increase in the number of arginine residues, APR aggregation propensity, compatibility with the aggregate core structure, adopting β-turn-promoting motifs, as well as introducing disulphide bonds, are able to improve Pept-in antimicrobial potency. The most successful modifications decreased P2 MIC from 12.5 μg/mL to 3.13 μg/mL. We next sought to investigate whether these structure modifications have a synergistic effect for improving Pept-in antimicrobial potency. However, combining multiple structure modifications did not result in a MIC value lower than 3.13 μg/mL (S9 Table), demonstrating a lack of synergistic effects for improving Pept-in antimicrobial potency over a certain threshold.

We have shown that increasing Pept-in arginine number or aggregation propensity can improve antimicrobial potency for both P2 and P33, suggesting that these antimicrobial beneficial structure modifications can sometimes be directly translated between Pept-ins with different APRs. Next, we further examined whether the beneficial effect of Pept-in structure modifications on antimicrobial potency can be directly applied between P2 with a different number of arginines. For this purpose, we assessed whether linker modification on P2-4R (with mutations R10A and R20A), which showed a 2-fold MIC increase in *E*. *coli* BL21 compared to P2 (S10 Table), will have the same impact as observed for P2. Similar to P2, flexible linkers did not improve P2-4R antimicrobial potency (S10 Table). Although these β-turn-promoting linkers all improved P2 antimicrobial potency (S7 Table), only GV (P2_4R_GV) improved P2-4R antibacterial activity with a MIC decrease from 25 μg/mL to 6.3 μg/mL (S10 Table). Additionally, a group of helix-promoting linkers [44] improved P2-4R activity to the same level as P2, such as P2_4R_L (S10 Table). The inconsistent effect of β-turn-promoting linkers on P2-4R and P2 suggests that the beneficial effect of structure modifications cannot always be directly translated between different Pept-ins, especially when the number of gatekeepers is different.

In the absence of polyP, P2-4R formed mainly β-sheet structures with a strong maximum around 1627 cm^-1^, while P2-4R-GV and P2-4R-L formed mostly α-helix with a sharp peak near 1652 cm^-1^ (Fig 4H). In the presence of polyP, P2-4R and P2-4R-GV formed fibrils, while P2-4R-L formed both fibrils and amorphous aggregates (S5D Fig). The fibrils formed by P2-4R consists of predominantly β-sheets with a sharp peak near 1625 cm^-1^, while P2-4R-GV and P2-4R-L mainly formed the non-classical α-helical aggregates [45] as indicated by the sharp peak near 1652 cm^-1^ (Fig 4I). Taken together, the data show that the ability of Pept-ins to form large aggregates in the presence of polyP appears to correlate to their antimicrobial potency, while the morphological feature (amorphous aggregates or ordered fibrils) and secondary structure (β-sheet or α-helix) do not.

### Improved antimicrobial activity against laboratory-derived P2-resistant strains and clinically isolated multidrug-resistant strains

**Fig 5:**
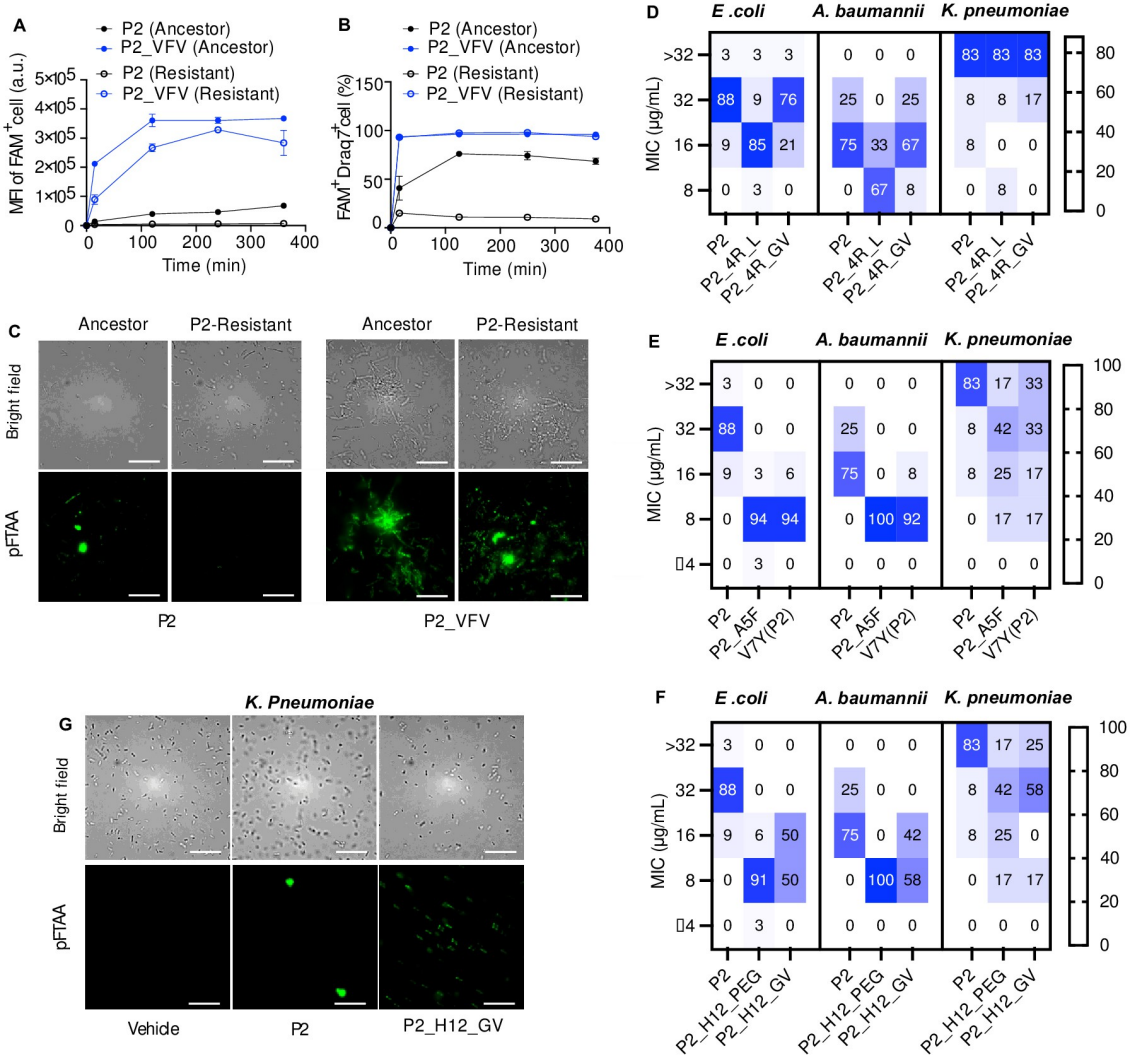
Improved antimicrobial activity against laboratory-derived P2-resistant strains and clinically isolated multi-drug resistant strains. **A-B:** Flow cytometry analysis of ancestors and P2-resistant *E*. *coli* treated with FAM-labelled Pept-ins for different time points at the concentration of FAM-P2-MIC from three independent experiments. FAM MFI of FAM positive cell population (**A**), and the percentage of FAM and Draq7 positive cells (**B**) when treated with P2 and P2-VFV. Error bars represent SEM (n = 3). **C**: SIM images of ancestors and P2-resistant strains treated with P2 and P2-VFV for 4 h at the concentration of FAM-P2-MIC. Amyloid-specific dye pFTAA was incubated with bacteria for 1.5 h. **D-F** is associated with S12 Table, the number of tested isolates for *E*. *coli*, *A*. *baumannii*, and *K*. *pneumoniae* is 34, 12, and 12, respectively. **D**: The percentage of *E*. *coli*, *A*. *baumannii*, *or K*. *pneumoniae* isolates which had a MIC at >32, 32, 16 or 8 μg/mL for P2, P2_4R_L and P2_4R_GV. The colours white to blue in the heatmap indicate an increased percentage of isolates. **E**: The percentage of *E*. *coli*, *A*. *baumannii*, *or K*. *pneumoniae* isolates which had a MIC at >32, 32, 16, 8, or ≤4 μg/mL for P2, P2_A5F and V7Y (P2). The colours white to blue in the heatmap indicate an increased percentage of isolates. **F**: The percentage of *E*. *coli*, *A*. *baumannii*, *or K*. *pneumoniae* isolates which had a MIC at >32, 32, 16, 8, or ≤4 μg/mL for P2, P2_H12_PEG and P2_H12_GV. The colours white to blue in the heatmap indicate an increased percentage of isolates. **G:** SIM of *K*. *pneumoniae* treated by P2 and P2_H12_GV at 32 ug/mL for 2 h. Amyloid-specific dye pFTAA was incubated with bacteria for 1.5 h. Scale bar: 10 μm.

In a previous study, we generated a P2-resistant strain by subculturing the hypermutable *E*. *coli* XL1-Red, which has a 5 000-fold higher mutation rate than wildtype bacteria, at the sub-MIC concentration of P2 for 27 days. This P2-resistant strain was able to keep P2 intracellular accumulation at a very low concentration and thereby causing a P2 MIC increase from 6.25 μg/mL (for ancestor strain) to 200 μg/mL (for P2 resistant strain) [23]. The MIC of a few categories of P2 variants were determined to investigate whether P2 antimicrobial activity can be restored against P2-resistant bacteria via structure modification. The P2-resistant strain showed a MIC value lower than 200 μg/mL for the variants with a β-turn promoting linker, a disulphide bond or increased APR aggregation propensity (S11 Table). P2_H12_GV and P2_VFV were especially active against the P2-resistant strain (with a MIC at 6.25 μg/mL), indicating that structure modification is indeed able to restore P2 activity against P2-resistant bacteria (S11 Table). The amount of intracellularly accumulated P2_VFV in P2-resistant strains was much higher than P2 (Fig 5A), which was sufficient to induce cell death (FAM^+^Draq7^+^ cells) (Fig 5B). Additionally, in contrast to the absence of protein aggregation events in P2-resistant bacteria upon P2 treatment, protein aggregation events were observed in the resistant bacteria when treated by P2_VFV, as demonstrated by the positive staining of amyloid specific dye pFTAA [27] (Fig 5C). The data here suggest that the mechanisms governing Pept-in uptake in bacteria can be altered via structure modification and thus sheds light on the future clinical application of Pept-ins if encountering resistance.

To address the emerging antibiotic resistance issue, it is not only required to develop active antibiotics against wildtype bacteria, but also against the clinically isolated multidrug-resistant strains. Therefore, we determined whether P2 variants with a 4-fold lower MICs (P2_A5F, V7Y (P2), P2_H12_GV, P2_H12_PEG) (S12 Table) will also show improved antimicrobial activity against these multidrug-resistant strains. Since a high net positive charge could carry inherent developability challenges [46, 47], we further explored the therapeutic potential of P2 variants with reduced net charge (P2_4R_GV and P2_4R_L) (S12 Table) by determining their antimicrobial activity against these clinically isolated multidrug-resistant strains. The mode of action of these derivatives remains to be inducing protein aggregation in bacteria as shown by the formation of IBs stained by pFTAA after Pept-in treatment (P2, P2-4R-GV, P2_A5F, V7Y (P2), P2_H12_GV, P2_H12_PEG) (S6A Fig) and the presence of a large number of proteins with different molecular weights in purified IBs as shown by the Coomassie staining (S6B Fig), in contrast to the absence of pFTAA staining (S6A Fig) and the presence of only two faint protein bands (S6B Fig) for buffer and control peptide treated bacteria. For clinically isolated multidrug-resistant strains, in addition to the 34 clinical isolates of *E*. *coli ATCC*, we added 12 *Acinetobacter baumannii (A*. *baumannii)* isolates *and* 12 *Klebsiella pneumoniae (K*. *pneumoniae)* isolates to broaden the selection of the bacterial spectrum. Eight proteins containing a homologous APR to P2 APR with 1 amino acid mismatch were identified in the gram-negative *A*. *baumannii* (S13 Table), and P2 shows a MIC at 16 μg/mL against wildtype *A*. *baumannii*. However, P2 shows little activity (MIC > 32 or >128 μg/mL, depending on the maximum tested concentration) against the wildtype gram-negative *K*. *pneumoniae*, although 21 proteins containing a homologous APR to P2 with 1 amino acid mismatch were identified in its proteome (S13 Table).

For P2 with four arginine residues, we have shown that P2_4R_GV showed a 2-fold lower MIC than P2 and P2_4R_L against *E*. *coli* BL21 (S10 Table). However, when tested on the clinical isolates of *E*. *coli* ATCC and *A*. *baumannii*, P2_4R_L demonstrated a 2-fold lower MIC than P2 and P2_4R_GV (Fig 5D, S12 Table). While 85% of *E*. *coli ATCC* and 67% of *A*. *baumannii* isolates showed a MIC at 16 μg/mL and 8 μg/mL for P2-4R_L, the majority of *E*. *coli ATCC* and *A*. *baumannii* isolates showed a MIC at 32 μg/mL and 16 μg/mL for P2 and P2_4R_GV (Fig 5D, S12 Table). Additionally, P2, P2_4R_L and P2_4R_GV all displayed a MIC at > 32 μg/mL against *K*. *pneumoniae* (Fig 5D, S12 Table), which was the highest concentration tested in the screen. These results collectively demonstrate that P2 with four arginine residues remain active in multidrug-resistant clinical isolates of *E*. *coli ATCC* and *A*. *baumannii*, although the effect of linker on antibacterial activity is strain-dependent.

The enhanced antimicrobial activity of P2 variants with increased compatibility to the aggregate core structure (P2_A5F, V7Y (P2)) was further confirmed in the clinical isolates of all three strains. Most of the *E*. *coli* ATCC (88%) and *A*. *baumannii* (75%) isolates showed a P2 MIC at 32 μg/mL and 16 μg/mL, respectively (Fig 5E, S12 Table). However, the majority of *E*. *coli* ATCC (94% and 94%) and *A*. *baumannii* (100% and 92%) showed a 4-fold and 2-fold lower MIC against P2-A5F and V7Y (P2) compared to P2 (Fig 5E, S12 Table). A P2 MIC at > 32 μg/mL (83%) was observed in the majority of the *K*. *pneumoniae* isolates; however, the MIC at > 32 μg/mL was only observed in a minority of isolates when treated by P2-A5F (17%), V7Y (P2) (33%) (Fig 5E, S12 Table). Instead, the MIC in the lower range (32, 16, 8 μg/mL) was observed more frequently for P2-A5F, V7Y (P2), when compared to P2 (Fig 5E, S12 Table). Similarly, the improved antibacterial activity of P2 variants with a disulphide bond formation (P2_H12_PEG, P2_H12_GV) was observed in the clinical isolates of three strains as well (Fig 5F, S12 Table). We also observed that the improved antibacterial activity of P2_H12_GV against *K*. *pneumoniae* is accompanied by the formation of aggregates in a higher percentage of bacteria (Fig 5G), further confirming that Pept-in mode of action is achieved via inducing protein aggregation in bacteria. Therefore, structure variation of Pept-ins, such as forming a disulphide bond, is able to improve its antibacterial activity across different strains, which has been previously observed [48–50].

### Enhancing antimicrobial potency while minimising toxicity to mammalian cells

In addition to Pept-in antimicrobial potency, its toxicity against mammalian cells is also critical for determining its therapeutic potential. Thus, we determined the toxicity effect of P2 and eleven derivatives with enhanced antimicrobial activity (S14 Table) representing the following categories to human embryonic kidney (HEK 293T) cells, (i) with increased aggregation propensity (P2_IM, P2_M) or increased compatibility with the aggregate core structure (P2_A5F, V7Y (P2)), (ii) adopting β-turn-promoting motifs or a forming disulphide bond (P2_pG, P2_GV, P2_H12_GV, P2_H12_PEG), (iii) altered amount of arginine (P2_4R_GV, P2_4R_L, P2_RR). Additionally, the toxicity of P2_VFV and P2_4R, with a similar and lower antimicrobial activity as P2, was also evaluated.

**Fig 6:**
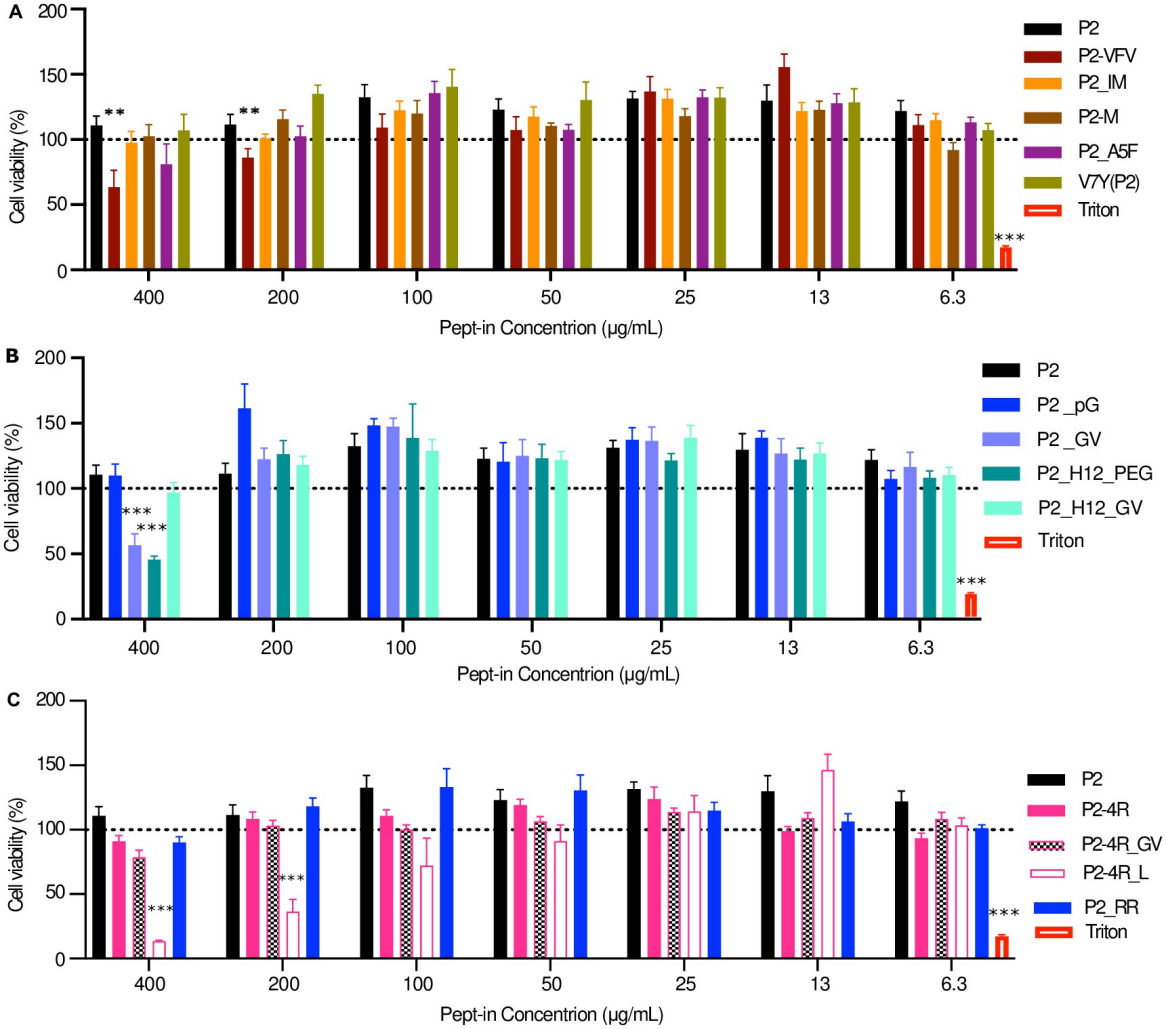
Enhancing antimicrobial potency while exhibiting no toxicity to mammalian cells. **A-B:** Concentration-dependent toxicity by CellTiter Blue assay on HEK 293T cells for P2 and derivatives with improved antimicrobial potency. **A:** Derivatives with increased aggregation propensity or compatibility to the aggregate core structure. **B:** Derivatives of linker modification or cysteine addition. **C**: Derivatives of altered number of arginine residues. Error bars represent SEM. Two-tailed Student t-test was performed for calculating statistical significance between the mean of P2 variant and P2 at the corresponding concentration (n = at least 6). Asterisks indicating the level of the p-value centred over the error bar mean: *p < 0.05, **p < 0.01, ***p < 0.001, and ****p < 0.0001.

Nine out of the thirteen P2 derivatives remained non-toxic at all tested concentrations (Fig 6A, 6B and 6C), demonstrating that these structural modifications are efficient to enhance the Pept-in therapeutic window by improving antimicrobial potency while remaining non-toxic to mammalian cells. However, P2-VFV with the highest TANGO score showed higher toxicity at 400 μg/mL and 200 μg/mL than P2 (Fig 6A). Therefore, the aggregation propensity of Pept-ins needs to be carefully assessed, since if the aggregation propensity is too high, its beneficial effect on improving antimicrobial potency disappears and a higher toxicity effect on mammalian cells can be induced. Notably, P2_GV, P2_H12_PEG (Fig 6B) and P2-4R-L (Fig 6C) induced toxicity effects (Fig 6B) at a higher concentration, indicating that linker modification could affect its toxicity on mammalian cells, possibly due to an altered secondary structure.

## Discussion

Although Pept-ins demonstrate considerable promise as an antibiotic strategy, investigation into the detailed mechanism of action and optimisation of Pept-in structure is required to increase their therapeutic potential. By studying the structure-activity relationship of Pept-ins, we addressed the importance of each Pept-in structure characteristic in mediating antimicrobial activity and identified approaches for enhancing Pept-in antimicrobial potency while minimising toxicity to mammalian cells *in vitro*.

Results here confirmed that Pept-in uptake is indeed positively correlated with Pept-in antimicrobial potency and the uptake efficiency is determined by multiple factors, such as the number of arginines (Fig 1D and 1E). We have previously shown that the electrostatic attraction between the negatively charged bacterial membrane and the positively charged Pept-ins is critical for Pept-in uptake [23]. The importance of electrostatic attraction for the initial interaction with the bacterial cell wall and the subsequent interaction with the lipid membrane has been addressed for cationic AMPs as well [1, 23, 25, 51]. Although anionic AMPs such as dermcidin have been reported, it requires Zn^2+^ for the interaction with the bacterial membrane [52]. Interestingly, charge distribution only affected Pept-in antimicrobial activity when the net charge is at the lower range (< +5) (Fig 1A and 1B, S1 and S2 Tables). Previous studies have shown that charge distribution affects AMP pore formation [53], antibacterial potency [54] and cell selectivity [55]. When the amount of charge is the same, modifying charge distribution affects peptide amphipathicity, which could influence the interaction with membrane and thereby affecting Pept-in activity. Furthermore, arginine is more efficient in promoting Pept-in uptake than histidine, likely because an acid environment is required for the protonation of histidine-rich peptides [56–58]. Similarly, AMPs enriched with histidine are less frequently found and their activity, such as Gad 5 [57] or piscidins [59], is often pH-dependent.

Pept-in APR aggregation propensity is another factor that strongly affects its uptake efficacy (Fig 2D and 2F). Increased APR aggregation propensity promotes Pept-in uptake, possibly due to the observation that aggregated or multimeric peptides can achieve a high surface concentration around bacterial cell wall [60]. Additionally, increased Pept-in APR aggregation propensity can be contributed by a high hydrophobicity, which is also an important driving force for peptides to cross bacterial membrane [60].

Data here further demonstrated that a higher amount (Figs 1D, 1E, 1G, 1H, 2D, 2F, 3D and 4D), as well as a faster kinetic profile of protein aggregation events induced by Pept-ins in bacteria (Figs 1F, 1I, 2E, 2G, 3E, 3G and 4E), are associated with a higher antimicrobial potency. A higher amount of protein aggregation events, especially when the sequestered proteins are involved in more diverse biological pathways, will have a more devastating effect on bacterial viability. While a faster kinetic profile of inducing protein aggregation in bacteria will contribute to a more overwhelming effect of the bacterial protein quality control system. Enhancing Pept-in efficiency in inducing protein aggregation can be achieved by improving Pept-in uptake or optimizing the aggregate core structure.

Since the decreased aggregation event in bacteria is mostly accompanied by a reduced uptake, it is, therefore, difficult to conclude whether this Pept-in modification directly caused the decreased protein aggregation events. However, P2 with lysine as gatekeepers lost its antimicrobial activity despite a fast and efficient intracellular uptake (Fig 1G and 1H), accompanied by the absence of protein aggregation events in bacteria (Fig 1I). Therefore, the observation that Pept-in uptake and the subsequent ability to induce protein aggregation in bacteria are the determining factors for Pept-in antibacterial potency, supports the conclusion that the mode of action of Pept-in is mainly achieved intracellularly by inducing protein aggregation and subsequently disrupting bacterial proteostasis.

Our results also demonstrated the ability of Pept-ins to stay in the soluble state in solution but being able to polymerise into large insoluble aggerates in the presence of aggregation-promoting molecules is critical for its antimicrobial activity (Figs 1C, 2C, 3H and 4C). This makes sense since protein aggregation behaviour can be different between *in vitro* and in bacteria. Elements that contribute to this difference are the highly crowded environment in cytoplasm or periplasm [61, 62] and the presence of molecules in bacteria that are able to promote protein aggregation, such as polyP [31], lipopolysaccharides and lipoteichoic acid [63]. Therefore, staying in the soluble state could be beneficial for Pept-ins to interact with bacteria, while being able to eventually aggregate upon entering bacteria could be essential for inducing protein aggregation events in bacteria.

The active antimicrobial Pept-ins could form either β-sheet (Figs 1K, 2I, 3J and 4G) or α-helix (Fig 4I) enriched aggregates in the presence of polyP, suggesting that Pept-in uptake and antimicrobial efficacy could be associated with the secondary structure to a lesser extent compared to the net charge and the aggregation propensity. Similarly, while the hydrophobic and electrostatic interaction are two driving forces that promote AMPs to interact with bacterial membrane [60], the effect of secondary structure on AMP antibacterial potency is not universal. Amphipathic AMPs with an α-helical structure sometimes show more potent activity than the wildtype with random structure [64], such as temporin-Ali [65], indicating the beneficial role of α-helix. While other studies suggest that β-hairpin structures improve AMP antimicrobial potential, for example, the most potent natural AMPs are β-hairpin peptides such as horseshoe crab polyphemusin I, pig protegrin [66] and defensins [64]. However, another group of studies suggest that AMP activity is independent of secondary structure but rather the amphipathicity [67, 68], for example, the conversion of pardaxin from an α-helix to β-sheets by the incorporation of D-amino acid residues showed similar antimicrobial activity [68]. To further demonstrate the relationship between Pept-in secondary structure and activity, future studies should investigate the structural features induced by membrane-peptide interaction, rather than the structure itself in solution.

In summary, structural modifications such as balancing the number of arginine residues, increasing Pept-in aggregation propensity, optimizing the aggregate core structure, adopting β-turn linkers, or forming a disulphide bond, can enhance Pept-in antimicrobial potency. These structural modifications can be adopted as general rules for future Pept-in design or for Pept-in optimisation against clinically isolated multidrug-resistant strains. This will improve the success rate, decrease the required time and improve the therapeutic potential of designing Pept-ins against a broader range of strains. However, we were not able to achieve a P2 MIC lower than 3.1 μg/mL against *E*. *coli*. Since Pept-in uptake and the subsequent protein aggregation events seem to be the determining factor for Pept-in antimicrobial potency, future studies could explore strategies to enhance Pept-in uptake and thereby further improving its potency.

## Star ★ methods

Detailed methods are provided in the online version of this paper and include the following:

- KEY RESOURCES TABLE
- RESOURCE AVAILABILITY
  - **Lead Contact**
  - **Materials Availability**
- EXPERIMENTAL MODEL AND SUBJECT DETAILS
- METHOD DETAILS

### Key resources table

**Table:** 

**REAGENT or RESOURCE**	**SOURCE**	**IDENTIFIER**
**Bacterial and Virus Strains**		
*E*. *coli* BL21	NEB	N/A
*E*. *coli* XL1 Red	Agilent	200129
P2 resistant XL1 Red	[23]	N/A
*E*. *coli* ATCC 25922 (Clinical isolates)	IHMA	N/A
*P*. *aeruginosa* ATCC 27853(Clinical isolate))	IHMA	N/A
*K*. *pneumoniae* (Clinical isolates)	IHMA	N/A
**Chemicals, Peptides, and Recombinant Proteins**		
Peptide	Ordered from GenScript or synthesised in house	N/A
HS-169	[26]	N/A
pFTAA	[27]	N/A
Draq7	BioStatus	Cat #: DR71000
PolyP	Merck	Cat #: 305553
Mueller-Hinton Broth	Midland Scientific	Cat #: 275730
DMSO	Merck	Cat #: D8418
Precision Plus Protein™ All Blue Prestained Protein Standards	Bio-Rad	Cat#: 1610373
**Critical Commercial Assays**		
CellTiter-Blue^®^ Reagent	Promega	Cat #: G808A
**Experimental models: Cell line**		
HEK 293T	ATCC	ATCC CRL-3216
**Software and Algorithms**		
GraphPad Prism 9.0	GraphPad Software, USA	https://www.graphpad.com
FlowJo™ Software 10.7.1	Ashland, USA	https://www.flowjo.com
FIJI 2.0.0-rc-68/1.52f	[69]	https://fiji.sc

### Resource availability

#### Lead contact

Further information and requests for resources and reagents should be directed to and will be fulfilled by the lead Contact, Frederic Rousseau (frederic.rousseau@kuleuven.bemailto:joost.schymkowitz@kuleuven.be).

#### Materials availability

Clinical isolates of *K*. *pneumoniae* used in this study are available from the Lead Contact with a completed Material Transfer Agreement.

### Experimental model and subject details

*E*. *coli* BL21, clinical isolates of *K*. *pneumoniae*, *laboratory-derived* P2 resistant *E*. *coli* XL1 Red, HEK 293T cells were cultured as described in the Method details.

## Method details

### Peptide preparation

Peptides were either synthesised in the Switch laboratory using standard solid-phase peptide synthesis (JPT, Berlin, Germany) or purchased from GenScript (Leiden, The Netherlands). Peptides with ≥ 90% purity were used in the downstream experiments. The lyophilised peptides were dissolved at 1–5 mg/ml in indicated buffer and sonicated for 5 min in a water sonication bath to make sure they were completely dissolved. The peptide stock was further diluted to desired experimental concentrations. For disulphide bond formation of cysteines, air oxidation was performed [70]. Briefly, the peptide was dissolved in DMSO and diluted with water (10% DMSO by volume). The oxidation experiment was performed at room temperature for about 17 hours. The completion of disulphide bond formation was confirmed by HPLC and LCMS.

### MIC determination

The peptide MIC was determined via the broth microdilution assay according to the EUCAST guideline, using 96-well polystyrene flat-bottom microtiter plates (BD Biosciences). Briefly, a single colony was inoculated in 5 mL Mueller-Hinton (MH) medium and incubated, while shaking at 37°C, to the end-exponential growth phase. The bacterial culture was subsequently diluted to 0.005 McFarland (McF) (~1.5x10^6^ CFU/mL) with fresh MH medium. Two-fold serial peptide-dilutions were prepared in 50 μL MH medium resulting in a concentration range of 0.75 to 100 μg/mL (at least three wells for each concentration). Afterwards, 50 μL of the freshly diluted bacterial suspension (0.005 McF) was mixed with different peptide concentrations in the 96-well plate, performed in triplicates. The plate was ultimately incubated at 37°C overnight without shaking for 17 hours. The MIC was determined as the lowest peptide concentration without visible bacterial growth.

### Flow cytometry analysis of peptide treated bacteria

Flow cytometry is used to determine peptide accumulation inside bacteria and its correlation with bacterial death. Briefly, end-exponential growth phase bacteria were washed twice with filtered PBS (7 000 rpm, 4 min) and diluted to 0.5 McF (1.5x10^8^ CFU mL^-1^) with PBS (pH 7.4). 1 mL bacteria suspension was treated with FAM-labelled peptide at the described concentrations and incubated at 37°C without shaking for the following time points: 15 min, 2 h, 4 h and 6 h. Treated bacteria were washed three times (7 000 rpm, 4 min) with PBS (pH 7.4) and resuspended in 200 μL PBS. If indicated, 1 μL of Draq7 (Biostatus) or 1 uL of HS-169 was added and incubated for 10 min at room temperature in the dark before acquisition. HS-169 instead of pFTAA was used to investigate protein aggregation for this experiment since pFTAA and FAM have overlapping emission spectra. Samples were either acquired on a Gallios Flow Cytometry (Beckman Coulter) or BD Fortessa X-20, data was analysed with Flowjo software version 10.6. Bacteria were first gated on forward-scatter (FSC) and side-scatter (SSC) and then followed by doublets exclusion. Median fluorescence intensity (MFI) is calculated using Flowjo, which sorts the fluorescence intensity of FAM^+^ cells from least to most and then selects the middle point. Maximum excitation/emission wavelength (FAM: 490/525 nm, Draq7: 633/697 nm, HS-169: 535/665 nm).

### pFTAA kinetic assay

DMSO and PBS (pH 7.4) were initially filtered with a 0.22 μM regenerated cellulose filter (Whatman USA). Peptides were dissolved with DMSO (10% v/v) in PBS (pH 7.4) to 2 mg/mL and subsequently diluted to 50 μM with PBS. 70 μL Peptide was incubated with pFTAA (0.5 μM) in a flat-bottom 96-well half-area microplate (Corning). The pFTAA fluorescence emission was recorded at 525–10 nm after excitation at 450 nm using a FLUOstar Omega plate reader (BMG labtech, Germany) for 8 hours.

### Transmission electron microscopy

Formvar film-coated 400-mesh copper grids (Agar Scientific Ltd., England) were first glow-discharged. 5 μl of each sample was taken from the end of the pFTAA kinetics experiment and adsorbed for 5 min on the grids. The grids were washed by contact with one drop of ultrapure water and then negatively stained by contact with one drop of uranyl acetate (2% w/v) for 1 min. The grids were washed by contact with 3 drops of ultrapure water and examined using a JEM-1400 transmission electron microscope (Jeol, Japan) at accelerating voltage 80 keV.

### Fourier-Transform infrared spectroscopy (FTIR)

DMSO and PBS (pH 7.4) were initially filtered with a 0.22 μM regenerated cellulose filter (Whatman USA). Peptides were dissolved with DMSO (6% v/v) in PBS (pH 7.4) to 1 mg/mL and left on the bench at room temperature for at least 8 hours before proceeding with the FTIR analysis. Peptides incubated with PolyP (0.25 mM) were left on the bench at room temperature for at least 16 hours before proceeding with the FTIR analysis. The FTIR spectrum of 30 μL peptide samples (n = 3) was recorded by averaging 60 scans in the range of 4000 cm^-1^ to 900 cm^-1^ at a resolution of 4 cm^-1^ using a FTIR spectrometer INVENIO (Bruker) coupled with an ATR (a diamond crystal). The normalisation of amide I region (1700 cm-1 to 1600 cm-1) was performed by dividing the absorbance of peptide samples to the vehicle at each corresponding wavenumber using Graphpad prism.

### TANGO score calculation

Aggregation propensity of APRs was obtained using TANGO [11], which predicts β-aggregation propensity of peptides and returns a TANGO score for each given APR sequence. For this analysis, TANGO parameters were set to pH 7.5, ionic Strength 0.01 M, temperature = 298 K.

### Super-resolution microscopy of peptide treated bacteria

Peptide treated bacteria were stained with amyloid-specific dye pFTAA (0.5 μM) and visualised with super-resolution microscopy (SIM) to confirm the presence of inclusion body formation in bacteria. Briefly end-exponential growth phase bacteria were washed twice with filtered PBS (7 000 rpm, 4 min) and diluted to 0.5 McF (1.5x10^8^ CFU mL^-1^) with PBS. 1 mL diluted bacteria suspension was treated with peptide at the described concentration for 2 h and followed by another 1.5 h treatment of 10 μL pFTAA at 37°C without shaking. If indicated, HS-169 was added and incubated for 10 min at room temperature in the dark. Bacteria samples were visualised by SIM and acquired images were analysed on the image processing software FIJI [69]. Maximum excitation/emission wavelength (pFTAA: 450/525 nm).

### IB purification and Coomassie staining of purified IBs

IB purification of treated bacteria and Coomassie staining of purified IBs were performed as previously described [12]. Briefly, 10 mL of overnight cultured *E*. *coli* BL21 was treated by peptide at MIC concentration for 2 hours. The treated bacteria pellets were washed (at 4°C for 30 min at 4,000 × g) with 10 mL buffer A and then resuspended in 10 mL buffer B. The bacteria were then lysed by passing through a Glen Creston Cell Homogenizer (pressure: 20,000–25,000 psi) three times and then sonicated with a Branson Digital sonifier 50/60 HZ. The lysed cells were centrifuged (at 4°C for 30 min at 11,000 × g), and the pellets were resuspended with buffer D. The suspension was sonicated another three times to ensure that the IBs were completely dissolved. After centrifugation, the purified IBs were solubilized in 1 mL buffer F. The purified IBs were loaded on SDS gel (4–15% Mini-PROTEAN^®^TGX™ Precast Protein Gels, 10-well, 30 μl well volume) and stained by Coomassie blue (R250). Precision Plus Protein™ All Blue Prestained Protein Standards (250–10 kD) were used as a reference for Coomassie blue staining.

### CellTiter Blue assay

HEK 293T cells (ATCC CRL-3216) were grown in DMEM medium, supplemented with 10% FBS, 1 mM sodium pyruvate, non-essential amino acids. 100 μL of 10 000 cells were grown in flat-bottom 96-well plates (Fischer Scientific) overnight until cells are attached to the plate. Peptides were dissolved with Mini-plasco Nacl 0.9% (B. Braun) to 2 mg/mL and diluted to indicated concentrations using cell culture medium. Cells were treated by adding 100 μL peptides to each well for about 16 hours. Cells lysed with 1% Triton X-100 was included as a positive control. The toxicity of the peptide treatments was evaluated using the CellTiter-Blue Cell Viability Assay according to the instructions of the manufacturer (Promega, USA). The metabolic activity of viable cells is measured by recording fluorescence intensity at 590 nm after excitation at 560 nm. Cell viability is determined by normalising the fluorescence intensity of peptide- or triton-treated cells to the vehicle-treated sample.

## Supporting information

S1 Fig Representative flow cytometry plots. **A:** The schematic representation of Pept-in design. **B**: Representative flow cytometry plots for Fig 1D–1F, gated on single cells showing FAM (peptide) and HS169 (aggregation) fluorescence after 15 min, 2 h and 4 h treatment of the corresponding peptide. **C**: Representative flow cytometry plots for Fig 1G–1I, gated on single cells showing FAM (peptide) and HS169 (aggregation) fluorescence after 15 min, 2 h and 4 h treatment of the corresponding peptide.
(DOCX)

S2 Fig pFTAA kinetics, representative TEM images. **A:** Time dependence of pFTAA fluorescence intensity of P2 derivatives (50 μM) with altered gatekeepers (Error bars represent SD, n = 9). **B:** Representative TEM images of P2 and derivatives from the end of the kinetic experiment of Fig 1C. The presence of fibrillar aggregates is indicated by a red arrow. **C**: The percentage of FAM positive cells when treated with P2 or its derivatives for different time points at the concentration of FAM-P2 MIC from three independent experiments (Error bars represent SEM, n = 9). **D**: Time dependence of pFTAA fluorescence intensity of P2 derivatives (50 μM) by modifying APR interaction. **E:** Representative TEM images of P2 and derivatives from the end of the kinetic experiment of Fig 3H. The presence of fibrillar aggregates is indicated by a red arrow.
(DOCX)

S3 Fig The effect of changing Tango score on Pept-in MIC, pFTAA kinetics, representative TEM images. **A:** MIC (left Y axis) against *E*. *coli* BL21 and Tango score (right Y axis) of P33 derivatives generated by alanine scanning of the APR region. The red asterisk indicates the mutation which led to a lower MIC than P33 while resulted in a decreased Tango score. This figure is associated with data from S2 Table. The red bars represent APR TANGO score, and the red line is the baseline of P33 APR Tango score. The blue squares represent MIC values, and the blue line is the baseline of P33 MIC. **B**: A non-linear negative correlation between P2 Tango score and P2 MIC analysed by Graphpad prism (correlation analysis, two-tailed). **C**: The percentage of FAM positive cells when treated with P2 or its derivatives for different time points at the concentration of FAM-P2 MIC from three independent experiments (Error bars represent SEM, n = At least 3). **D**: Time dependence of pFTAA fluorescence intensity of P2 derivatives (50 μM) by modifying Pept-in aggregation propensity (Error bars represent SD, n = At least 6). **E:** Representative TEM images of P2 and derivatives from the end of the kinetic experiment of Fig 2C.
(DOCX)

S4 Fig Representative flow cytometry plots. **A:** Representative flow cytometry plots for unstained bacteria, single colour control for FAM and HS169. **B**: Representative flow cytometry plots for Figs 2D–2G, 3F–3G, gated on single cells showing FAM (peptide) and HS169 (aggregation) at 15 min, 2 h and 4 h.
(DOCX)

S5 Fig pFTAA kinetics, representative flow cytometry plots and TEM images. **A**: The percentage of FAM positive cells when treated with P2 or its derivatives for different time points at the concentration of FAM-P2 MIC from three independent experiments (Error bars represent SEM, n = At least 6). **B**: Time dependence of pFTAA fluorescence intensity of P2 derivatives (50 μM) by adopting beta-turn promoting linkers (Error bars represent SEM, n = 9). **C:** Representative TEM images of P2 and derivatives from the end of the kinetic experiment of Fig 4C. **D:** Representative TEM images of aggregates formed by P2 and derivatives in the presence of PolyP (after 8 h incubation).
(DOCX)

S6 Fig Representative SIM images of peptide-treated bacteria and Coomassie blue SDS-PAGE of purified IBs. **A:** SIM images *of E*. *coli* BL21 treated by buffer, P2, V7Y (P2), P2_4R_GV, P2_A5F, P2_H12_GV, P2_H12-PEG, and control peptide for 2 h at the corresponding MIC concentration. Amyloid-specific dye pFTAA was incubated with bacteria for 1.5 h. The MIC of the control peptide (NANPGLGLALVPNANPGLGLALV) is > 100 μg/mL against *E*. *coli* BL21. Scale bar: 10 μm. For the lower panel, the bacteria in the small box are enlarged to have a better view of the formed IBs (stained by pFTAA). **B:** Representative Coomassie blue SDS-PAGE of IBs purified *from E*. *coli* BL21 treated by the buffer, P2, V7Y (P2), P2_4R_GV, P2_A5F, P2_H12_GV, P2_H12-PEG, and control peptide. The molecular weight marker is shown at the first lane.
(DOCX)

S1 Table MIC of P2 variants (gatekeeper modification). (DOCX)

S2 Table MIC of P33 variants (alanine scan). (DOCX)

S3 Table MIC of P2 and P33 variants (alanine scan). (DOCX)

S4 Table MIC of P2 variants (P2-WG truncation). (DOCX)

S5 Table MIC of P2 variants (aggregation propensity). (DOCX)

S6 Table MIC of P2 variants (APR affinity modulation). (DOCX)

S7 Table MIC of P2 variants (linker modification). (DOCX)

S8 Table MIC of P2 variants (disulphide bond formation). (DOCX)

S9 Table MIC of P2 variants (limited synergistic effects). (DOCX)

S10 Table MIC of P2 variants (4 arginine and linker modification). (DOCX)

S11 Table MIC of P2 variants against P2-resistant strain strain (E. coli XL1 Red). (DOCX)

S12 Table MIC of P2 variants against clinically isolated multi-drug resistant isolates. (DOCX)

S13 Table Proteins identified in *K*. *pneumoniae* and *A*. *baumannii* which contain the APR of P2 (GLGLALV) by allowing 1 mismatch. (DOCX)

S14 Table MIC of P2 variants used for toxicity study. (DOCX)
