# Supplementary figures and images for: Enhanced therapeutic window for antimicrobial Pept-ins by investigating their structure-activity relationship
Source: PLoS One. 2023 Mar 31;18(3):e0283674. doi: 10.1371/journal.pone.0283674 (PMC10065276; doi:10.1371/journal.pone.0283674)

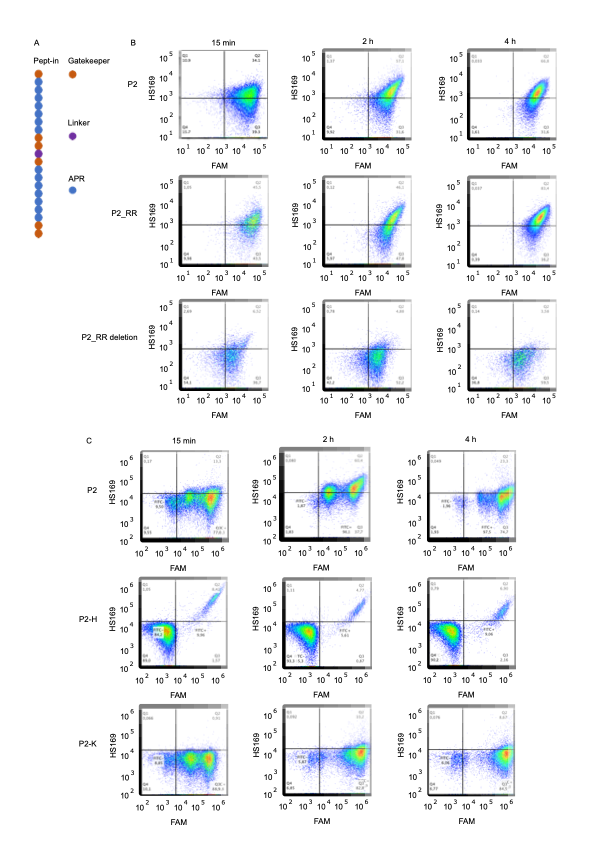

Supplement: S1 Fig — A: The schematic representation of Pept-in design. B: Representative flow cytometry plots for Fig 1D–1F, gated on single cells showing FAM (peptide) and HS169 (aggregation) fluorescence after 15 min, 2 h and 4 h treatment of the corresponding peptide. C: Representative flow cytometry plots for Fig 1G–1I, gated on single cells showing FAM (peptide) and HS169 (aggregation) fluorescence after 15 min, 2 h and 4 h treatment of the corresponding peptide. (DOCX) [file pone.0283674.s001.docx]

**
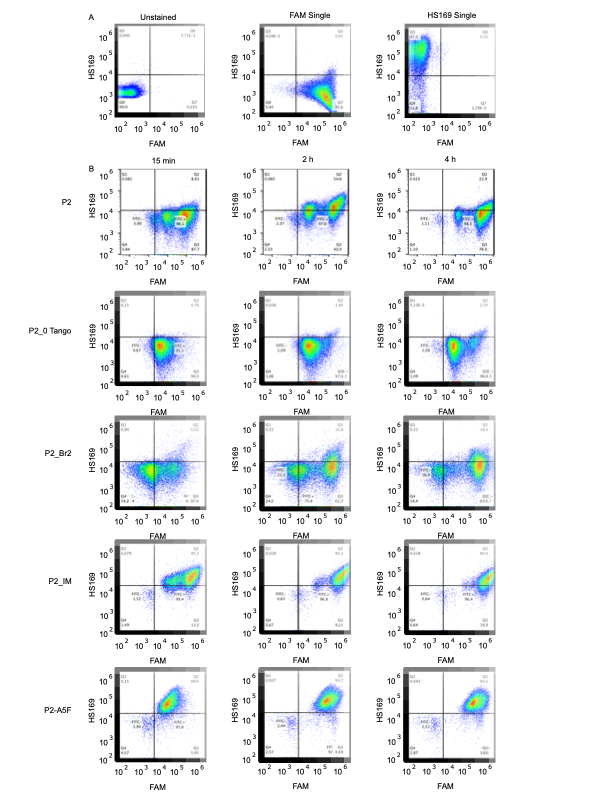
**

Supplement: S4 Fig — A: Representative flow cytometry plots for unstained bacteria, single colour control for FAM and HS169. B: Representative flow cytometry plots for Figs 2D–2G, 3F–3G, gated on single cells showing FAM (peptide) and HS169 (aggregation) at 15 min, 2 h and 4 h. (DOCX) [file pone.0283674.s004.docx]

**
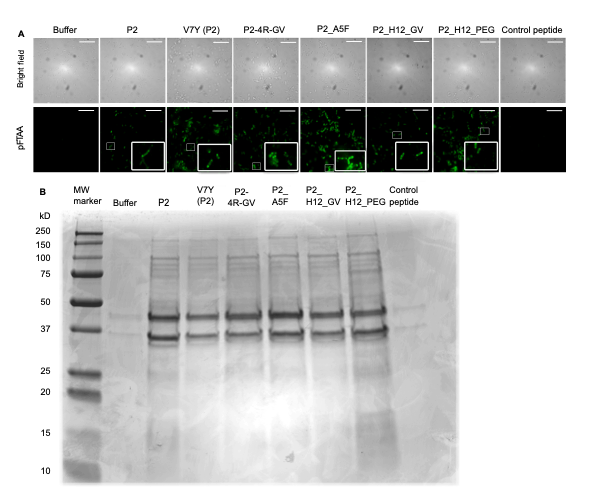
**

Supplement: S6 Fig — A: SIM images of E. coli BL21 treated by buffer, P2, V7Y (P2), P2_4R_GV, P2_A5F, P2_H12_GV, P2_H12-PEG, and control peptide for 2 h at the corresponding MIC concentration. Amyloid-specific dye pFTAA was incubated with bacteria for 1.5 h. The MIC of the control peptide (NANPGLGLALVPNANPGLGLALV) is > 100 μg/mL against E. coli BL21. Scale bar: 10 μm. For the lower panel, the bacteria in the small box are enlarged to have a better view of the formed IBs (stained by pFTAA). B: Representative Coomassie blue SDS-PAGE of IBs purified from E. coli BL21 treated by the buffer, P2, V7Y (P2), P2_4R_GV, P2_A5F, P2_H12_GV, P2_H12-PEG, and control peptide. The molecular weight marker is shown at the first lane. (DOCX) [file pone.0283674.s006.docx]
