# Supplementary material for: Enhanced therapeutic window for antimicrobial Pept-ins by investigating their structure-activity relationship
Source: PLoS One. 2023 Mar 31;18(3):e0283674. doi: 10.1371/journal.pone.0283674 (PMC10065276; doi:10.1371/journal.pone.0283674)
Supplement: S1 Table — (DOCX) [file pone.0283674.s007.docx]

**S1 Table . MIC of P2 variants (gatekeeper modification)**

| **Name** | **Sequence** | **Number of arginine** | | **BL21 MIC (μg/mL)** | **Modification** |
| --- | --- | --- | --- | --- | --- |
| P2 | RGLGLALVRRPRGLGLALVRR | | 6 | 12.50 |  |
| P2_GK_1 | RGLGLALVRPRGLGLALVRR | | 5 | 25.00 | Decrease of arginine number |
| P2_GK_2 | RGLGLALVRRPRGLGLALVR | | 5 | 25.00 |  |
| P2_R1A | AGLGLALVRRPRGLGLALVRR | | 5 | 25.00 |  |
| P2_R9A | RGLGLALVARPRGLGLALVRR | | 5 | 12.50 |  |
| P2_R10A | RGLGLALVRAPRGLGLALVRR | | 5 | 12.50 |  |
| P2_R12A | RGLGLALVRRPAGLGLALVRR | | 5 | 25.00 |  |
| P2_R20A | RGLGLALVRRPRGLGLALVAR | | 5 | 6.25 |  |
| P2_R21A | RGLGLALVRRPRGLGLALVRA | | 5 | 25.00 |  |
| P2-4R | RGLGLALVRAPRGLGLALVAR | | 4 | 25.00 |  |
| R9A, R20A | RGLGLALVARPRGLGLALVAR | | 4 | 50.00 |  |
| R9A, R21A | RGLGLALVARPRGLGLALVRA | | 4 | >100.00 |  |
| R10A, R21A | RGLGLALVRAPRGLGLALVRA | | 4 | >100.00 |  |
| P2_RR Deletion | RGLGLALVRPRGLGLALVR | | 4 | >100.00 |  |
| 1 | GLGLALVPGLGLALV | | 0 | >100.00 |  |
| P2_noGK_NANP3 | GLGLALVPNANPGLGLALV | | 0 | >100.00 |  |
| P2_noGK_NANP1 | GLGLALVPGLGLALVGNANPNANP | | 0 | >100.00 |  |
| 5 | QGLGLALVQQPQGLGLALVQQ | | 0 | >100.00 |  |
| P2_N | NGLGLALVNNPNGLGLALVNN | | 0 | >100.00 |  |
| P2_K | KGLGLALVKKPKGLGLALVKK | | 0 | >100.00 |  |
| P2_H | HGLGLALVHHPHGLGLALVHH | | 0 | >100.00 |  |
| P2_GK_8 | RRGLGLALVRPRGLGLALVRR | | 6 | 12.50 | Alteration of arginine distribution |
| P2_GK_9 | RGLGLALVRRPRRGLGLALVR | | 6 | 12.50 |  |
| P2_GK_10 | RRGLGLALVRPRRGLGLALVR | | 6 | 12.50 |  |
| P2_GK_4 | RRGLGLALVRRPRGLGLALVRR | | 7 | 6.25 | Increase of arginine number |
| P2_GK_5 | RGLGLALVRRRPRGLGLALVRR | | 7 | 6.25 |  |
| P2_GK_6 | RGLGLALVRRPRRGLGLALVRR | | 7 | 6.25 |  |
| P2_GK_7 | RGLGLALVRRPRGLGLALVRRR | | 7 | 12.50 |  |
| P2_RR addition | RRGLGLALVRRPRRGLGLALVRR | | 8 | 3.13 |  |
| P2-16R | RRRRRRRRNRGLGLALVRRPRGLGLALVRR | | 16 | 6.25 |  |
