# Supplementary material for: Enhanced therapeutic window for antimicrobial Pept-ins by investigating their structure-activity relationship
Source: PLoS One. 2023 Mar 31;18(3):e0283674. doi: 10.1371/journal.pone.0283674 (PMC10065276; doi:10.1371/journal.pone.0283674)
Supplement: S2 Table — (DOCX) [file pone.0283674.s008.docx]

**S2 Table . MIC of P33 variants (Alanine Scan)**

| **Name** | **Sequence** | **Number of arginine** | **BL21 MIC (μg/mL)** |
| --- | --- | --- | --- |
| P33 | PLGIAVALRRPRLGIAVALRR | 6 | 6.25 |
| P33_3R_1 | RLGIAVALPRLGIAVALR | 3 | 12.5 |
| P33_3R_2 | RLGIAVALRPLGIAVALR |  | 25.00 |
| P33_3R_4 | RLGIAVALRAPALGIAVALAR |  | 50.00 |
| P33_3R_3 | RLGIAVALARPALGIAVALAR |  | >100 |
| P33_4R_1 | RLGIAVALRPRLGIAVALR | 4 | 6.25 |
| P33_4R_4 | RLGIAVALRAPRLGIAVALAR |  | 12.50 |
| P33_4R_5 | RLGIAVALRRPALGIAVALAR |  | 12.50 |
| P33_4R_6 | RLGIAVALRRPRLGIAVALAA |  | 12.50 |
| P33_4R_2 | ALGIAVALRRPRLGIAVALAR |  | 25.00 |
| P33_4R_3 | RLGIAVALARPRLGIAVALAR |  | 50.00 |
| P33-R20A | RLGIAVALRRPRLGIAVALAR | 5 | 3.13 |
| P33-R1A | ALGIAVALRRPRLGIAVALRR |  | 6.25 |
| P33-R9A | RLGIAVALARPRLGIAVALRR |  | 6.25 |
| P33-R10A | RLGIAVALRAPRLGIAVALRR |  | 6.25 |
| P33-R12A | RLGIAVALRRPALGIAVALRR |  | 6.25 |
| P33-R21A | RLGIAVALRRPRLGIAVALRA |  | 6.25 |
