# Supplementary material for: Enhanced therapeutic window for antimicrobial Pept-ins by investigating their structure-activity relationship
Source: PLoS One. 2023 Mar 31;18(3):e0283674. doi: 10.1371/journal.pone.0283674 (PMC10065276; doi:10.1371/journal.pone.0283674)
Supplement: S3 Table — (DOCX) [file pone.0283674.s009.docx]

**S3 Table . MIC of P2 and P33 variants (Alanine Scan)**

| **Name** | **Sequence** | **Tango Score (APR)** | **BL21 MIC (μg/mL)** |
| --- | --- | --- | --- |
| P2 | RGLGLALVRRPRGLGLALVRR | 419.9 | 12.5 |
| P2_G2A | RALGLALVRRPRGLGLALVRR | 558.8 | 6.25 |
| P2_L3A | RGAGLALVRRPRGLGLALVRR | 419.9 | 12.50 |
| P2_G4A | RGLALALVRRPRGLGLALVRR | 581.2 | 6.25 |
| P2_L5A | RGLGAALVRRPRGLGLALVRR | 149.7 | 50.00 |
| P2_L7A | RGLGLAAVRRPRGLGLALVRR | 150.0 | 50.00 |
| P2_V8A | RGLGLALARRPRGLGLALVRR | 104.2 | 25.00 |
| P2_G13A | RGLGLALVRRPRALGLALVRR | 558.8 | 12.50 |
| P2_L14A | RGLGLALVRRPRGAGLALVRR | 419.9 | 25.00 |
| P2_G15A | RGLGLALVRRPRGLALALVRR | 581.2 | 12.50 |
| P2_L16A | RGLGLALVRRPRGLGAALVRR | 149.7 | 50.00 |
| P2_L18A | RGLGLALVRRPRGLGLAAVRR | 150.0 | 50.00 |
| P2_V19A | RGLGLALVRRPRGLGLALARR | 104.2 | 25.00 |
| P33 | RLGIAVALRRPRLGIAVALRR | 587.4 | 6.25 |
| P33-L2A | RAGIAVALRRPRLGIAVALRR | 450.9 | 12.50 |
| P33-G3A | RLAIAVALRRPRLGIAVALRR | 668.4 | 6.25 |
| P33-I4A | RLGAAVALRRPRLGIAVALRR | 279.4 | 12.50 |
| P33-V6A | RLGIAAALRRPRLGIAVALRR | 270.7 | 6.25 |
| P33-8LA | RLGIAVAARRPRLGIAVALRR | 425.2 | 6.25 |
| P33-L13A | RLGIAVALRRPRAGIAVALRR | 450.9 | 6.25 |
| P33-G14A | RLGIAVALRRPRLAIAVALRR | 668.4 | 6.25 |
| P33-I15A | RLGIAVALRRPRLGAAVALRR | 279.4 | 12.50 |
| P33-V17A | RLGIAVALRRPRLGIAAALRR | 270.7 | 6.25 |
| P33-L19A | RLGIAVALRRPRLGIAVAARR | 425.2 | 6.25 |
