# Supplementary material for: Enhanced therapeutic window for antimicrobial Pept-ins by investigating their structure-activity relationship
Source: PLoS One. 2023 Mar 31;18(3):e0283674. doi: 10.1371/journal.pone.0283674 (PMC10065276; doi:10.1371/journal.pone.0283674)
Supplement: S4 Table — (DOCX) [file pone.0283674.s010.docx]

**S4 Table. MIC of P2 variants (P2-WG truncation)**

| **Name** | **Sequence** | **Tango Score (APR)** | **ATCC MIC (μg/mL)** |
| --- | --- | --- | --- |
| P2-WG | WGRGLGLALVRRPRGLGLALVRR | 419.9 | 6.00 |
| Truncated 15 | WGRGLGLALVRRPRGLGLALVR | 419.9 | 25.00 |
| Truncated 14 | WGRGLGLALVRRPRGLGLALV | 419.9 | 25.00 |
| Truncated 13 | WGRGLGLALVRRPRGLGLAL | 75.3 | 50.00 |
| Truncated 12 | WGRGLGLALVRRPRGLGLA | 2.7 | >100.00 |
| Truncated 11 | WGRGLGLALVRRPRGLGL | 0 | >100.00 |
| Truncated 10 | WGRGLGLALVRRPRGLG | 0 | >100.00 |
| Truncated 9 | WGRGLGLALVRRPRGL | 0 | >100.00 |
| Truncated 8 | WGRGLGLALVRRPRG | 0 | >100.00 |
| Truncated 7 | WGRGLGLALVRRPR |  | >100.00 |
| Truncated 6 | WGRGLGLALVRRP |  | >100.00 |
| Truncated 5 | WGRGLGLALVRR |  | >100.00 |
| Truncated 4 | WGRGLGLALVR |  | >100.00 |
| Truncated 3 | WGRGLGLALV |  | >100.00 |
| Truncated 2 | WGRGLGLAL |  | >100.00 |
| Truncated 1 | WGRGLGLA |  | >100.00 |
