# Supplementary material for: Enhanced therapeutic window for antimicrobial Pept-ins by investigating their structure-activity relationship
Source: PLoS One. 2023 Mar 31;18(3):e0283674. doi: 10.1371/journal.pone.0283674 (PMC10065276; doi:10.1371/journal.pone.0283674)
Supplement: S5 Table — (DOCX) [file pone.0283674.s011.docx]

**S5 Table. MIC of P2 variants (aggregation propensity)**

| **Name** | **Sequence** | **Tango Score (APR)** | **BL21 MIC (μg/mL)** | **Comment** | **Modification** |
| --- | --- | --- | --- | --- | --- |
| P2 | RGLGLALVRRPRGLGLALVRR | 419.9 | 12.50 |  |  |
| P2_zero_tango_5 | RGLGLDLVRRPRGLGLDLVRR | 0.0 | >100.00 | APR modification | Decrease aggregation propensity |
| P2_zero_tango_2 | RGLGLADVRRPRGLGLADVRR | 0.0 | >100.00 |  |  |
| P2_zero_tango_3 | RGLGLAHHRRPRGLGLAHHRR | 0.0 | >100.00 |  |  |
| P2_zero_tango_1 | RGLGLAPVRRPRGLGLAPVRR | 0.0 | >100.00 |  |  |
| P2_zero_tango_4 | RGLGLALPRRPRGLGLALPRR | 0.0 | >100.00 |  |  |
| GlucNAc - P2 | N(GlcNAc)GRGLGLALVRRPRGLGLALVRR |  | >100.00 | Glycolysation |  |
| P2_Br1 | NANPGRGLGLALVRRPRGLGLALVRR |  | 50.00 |  |  |
| P2_Br2 | EPQSGRGLGLALVRRPRGLGLALVRR |  | >100.00 |  |  |
| P2_Br3 | DEPQSGRGLGLALVRRPRGLGLALVRR |  | >100.00 |  |  |
| P2_Br4 | SQEQSPNGRGLGLALVRRPRGLGLALVRR |  | >100.00 |  |  |
| P2_Br5 | QPNSEQSGRGLGLALVRRPRGLGLALVRR |  | >100.00 |  |  |
| P2_Br6 | NSPEQSQGRGLGLALVRRPRGLGLALVRR |  | >100.00 | Entropic bristles |  |
| P2_Br7 | NAYPGRGLGLALVRRPRGLGLALVRR |  | 100.00 |  |  |
| P2_Br8 | EPWSGRGLGLALVRRPRGLGLALVRR |  | 50.00 |  |  |
| P2_Br9 | DEPFSGRGLGLALVRRPRGLGLALVRR |  | >100.00 |  |  |
| P2_Br10 | SQEFSPNGRGLGLALVRRPRGLGLALVRR |  | >100.00 |  |  |
| P2_Br11 | QPNSEFSGRGLGLALVRRPRGLGLALVRR |  | >100.00 |  |  |
| P2_Br12 | NSPEWQSGRGLGLALVRRPRGLGLALVRR |  | >100.00 |  |  |
| P2_M | RMGLGLALVRRPRGLGLALVRR | 419.9 | 3.13 | APR mpdification | Increase aggregation propensity |
| P2_Y | RYGLGLALVRRPRGLGLALVRR | 495.1 | 6.25 |  |  |
| P2_L | RLGLGLALVRRPRGLGLALVRR | 561.1 | 6.25 |  |  |
| P2_TY | RTYGLGLALVRRPRGLGLALVRR | 514.7 | 6.25 |  |  |
| P2_TTY | RTTYGLGLALVRRPRGLGLALVRR | 500.5 | 6.25 |  |  |
| P2_F | RFGLGLALVRRPRGLGLALVRR | 654.6 | 6.25 |  |  |
| P2_TL | RTLGLGLALVRRPRGLGLALVRR | 591.2 | 6.25 |  |  |
| P2_TF | RTFGLGLALVRRPRGLGLALVRR | 698.1 | 6.25 |  |  |
| P2_TTL | RTTLGLGLALVRRPRGLGLALVRR | 583.5 | 12.50 |  |  |
| P2_TTF | RTTFGLGLALVRRPRGLGLALVRR | 709.4 | 6.25 |  |  |
| P2_IM | RIMGLGLALVRRPRGLGLALVRR | 738.8 | 6.25 |  |  |
| P2_LI | RLIGLGLALVRRPRGLGLALVRR | 849.7 | 6.25 |  |  |
| P2_VFV | RVFVGLGLALVRRPRGLGLALVRR | 987.9 | 12.50 |  |  |
| P2_VFV_VFV | RVFVGLGLALVRRPRVFVGLGLALVRR | 987.9 | 12.50 |  |  |
|  |  |  |  |  |  |
