# Supplementary material for: Enhanced therapeutic window for antimicrobial Pept-ins by investigating their structure-activity relationship
Source: PLoS One. 2023 Mar 31;18(3):e0283674. doi: 10.1371/journal.pone.0283674 (PMC10065276; doi:10.1371/journal.pone.0283674)
Supplement: S6 Table — (DOCX) [file pone.0283674.s012.docx]

**S6 Table. MIC of P2 variants (APR affinity modulation)**

| **Name** | **Sequence** | **Tango Score (APR)** | **BL21 MIC (μg/mL)** | **Modification** |
| --- | --- | --- | --- | --- |
| P2 | RGLGLALVRRPRGLGLALVRR | 419.9 | 12.50 |  |
| V7S (P2) | RGLGLALSRRPRGLGLALSRR​ | 44.4 | 100.00 | Incompatible |
| A5G (P2) | RGLGLGLVRRPRGLGLGLVRR​ | 116.2 | 25.00 |  |
| V7T (P2) | RGLGLALTRRPRGLGLALTRR​ | 85.5 | 50.00 |  |
| L6G (P2) | RGLGLAGVRRPRGLGLAGVRR​ | 19.9 | 100.00 |  |
| L6P (P2) | RGLGLAPVRRPRGLGLAPVRR | 0.0 | 100.00 |  |
| L6D (P2) | RGLGLADVRRPRGLGLADVRR | 0.0 | >100.00 |  |
| A5W (P2) | RGLGLWLVRRPRGLGLWLVRR​ | 559.5 | 12.50 | More comparable |
| A5F (P2) | RGLGLFLVRRPRGLGLFLVRR​ | 591.6 | 12.50 |  |
| A5L (P2) | RGLGLLLVRRPRGLGLLLVRR​ | 571.2 | 12.50 |  |
| A5M (P2) | RGLGLMLVRRPRGLGLMLVRR​ | 464.5 | 6.25 |  |
| A5I (P2) | RGLGLILVRRPRGLGLILVRR​ | 589.5 | 6.25 |  |
| A5Y (P2) | RGLGLYLVRRPRGLGLYLVRR​ | 547.5 | 6.25 |  |
| V7Y (P2) | RGLGLALYRRPRGLGLALYRR​ | 241.0 | 3.13 |  |
| P2_A5W | RGLGLALVRRPRGLGLWLVRR​ | 559.5 | 6.25 |  |
| P2_A5F | RGLGLALVRRPRGLGLFLVRR​ | 591.6 | 3.13 |  |
| P2_A5L | RGLGLALVRRPRGLGLLLVRR​ | 571.2 | 6.25 |  |
| P2_A5M | RGLGLALVRRPRGLGLMLVRR​ | 464.5 | 6.25 |  |
| P2_A5I | RGLGLALVRRPRGLGLILVRR​ | 589.5 | 12.50 |  |
| P2_A5Y | RGLGLALVRRPRGLGLYLVRR​ | 547.5 | 6.25 |  |
