# Supplementary material for: Enhanced therapeutic window for antimicrobial Pept-ins by investigating their structure-activity relationship
Source: PLoS One. 2023 Mar 31;18(3):e0283674. doi: 10.1371/journal.pone.0283674 (PMC10065276; doi:10.1371/journal.pone.0283674)
Supplement: S7 Table — (DOCX) [file pone.0283674.s013.docx]

**S7 Table. MIC of P2 variants (linker modification)**

| **Name** | **Sequence** | **BL21 MIC (μg/mL)** | **Comment** |
| --- | --- | --- | --- |
| P2 | RGLGLALVRRPRGLGLALVRR | 12.50 | Rigid linker |
| P2_P11A | RGLGLALVRRARGLGLALVRR | 12.50 | Alanine mutation |
| P2_19 | RGLGLALVRRGGRGLGLALVRR | 12.50 |  |
| P2_14 | RGLGLALVRRGSRGLGLALVRR | 12.50 | Flexible linker |
| P2_16 | RGLGLALVRRGGSRGLGLALVRR | 12.50 |  |
| P2_15 | RGLGLALVRRGSGSRGLGLALVRR | 25.00 |  |
| P2_17 | RGLGLALVRRGGGSRGLGLALVRR | 25.00 |  |
| P2_GV | RGLGLALVRRGVRGLGLALVRR | 6.25 | β-turn-promoting linker |
| P2 _pG | RGLGLALVRRpGRGLGLALVRR | 6.25 |  |
| P2_ fP | RGLGLALVRRfPRGLGLALVRR | 6.25 |  |
| P2- pP | RGLGLALVRpPRGLGLALVRR | 6.25 |  |
