# Supplementary material for: Enhanced therapeutic window for antimicrobial Pept-ins by investigating their structure-activity relationship
Source: PLoS One. 2023 Mar 31;18(3):e0283674. doi: 10.1371/journal.pone.0283674 (PMC10065276; doi:10.1371/journal.pone.0283674)
Supplement: S8 Table — (DOCX) [file pone.0283674.s014.docx]

**S8 Table. MIC of P2 variants (Disulphide bond formation)**

| **Name** | **Sequence** | **BL21 MIC (μg/mL)** | | **Comment** |
| --- | --- | --- | --- | --- |
|  |  | **no SS bond** | **SS bond** |  |
| P2 | RGLGLALVRRPRGLGLALVRR | 12.50 | - |  |
| P2_var9 | RGLGLALVCRRPCRGLGLALVRR | 6.25 | 6.25 | Cysteine |
| P2_H6_V | RCLGLALVRRVRGLGLALVRRGSC | 6.25 | 6.25 |  |
| P2_H12_GV | RGLGLALVCRRGVCRGLGLALVRR | 6.25 | 3.13 | β-turn-promoting linker & Cysteine |
| P2_H12_FC | RGLGLALVCRRRFCRGLGLALVRR | 6.25 | 3.50 |  |
| P2_H12_TG | RGLGLALVCRRTGCRGLGLALVRR | 6.25 | 6.50 |  |
| P2_H12_PEG | RGLGLALVCRR(PEG)CRGLGLALVRR | 6.25 | 3.13 |  |
| P2_H12_pG | RGLGLALVCRRpGCRGLGLALVRR | 6.25 | 3.13 |  |
| P2_H12_fP | RGLGLALVCRRfPCRGLGLALVRR | 6.25 | 3.13 |  |
| P2_H6 | RCLGLALVRRVRGLGLALRRGSC | 12.5 | 6.25 |  |
| P2_H6_pG | CRGLGLALVRRpGRRGLGLALVRGSC | 12.5 | 6.25 |  |
| P2_H13_pG | CRGLGLALVRRpGRRGLGLALVRC | 3.13 | 6.25 |  |
