# Supplementary material for: Enhanced therapeutic window for antimicrobial Pept-ins by investigating their structure-activity relationship
Source: PLoS One. 2023 Mar 31;18(3):e0283674. doi: 10.1371/journal.pone.0283674 (PMC10065276; doi:10.1371/journal.pone.0283674)
Supplement: S9 Table — (DOCX) [file pone.0283674.s015.docx]

**S9 Table. MIC of P2 variants (limited synergistic effects)**

| **Name** | **Sequence** | **Comment** | **BL21 MIC (μg/mL)** |
| --- | --- | --- | --- |
| P2 | RGLGLALVRRPRGLGLALVRR |  | 12.50 |
| P2_VFV_H12 | RVFVGLGLALVCRRGVCRGLGLALVRR | Increased aggregation propensity &  β-turn-promoting linker &  Cysteine | 50.00 |
| P2_M_H12 | RMGLGLALVCRRGVCRGLGLALVRR |  | 6.25 |
| P2_MM_H12 | RMGLGLALVCRRGVCRMGLGLALVRR |  | 6.25 |
| P2_A5F_H12_GV | RGLGLALVCRRGVCRGLGLFLVRR | Increased interaction affinity  & β-turn-promoting linker  &  Cysteine | 12.50 |
| V7Y (P2)_H12 | RGLGLALVCRRGVCRGLGLALYRR​ |  | 12.50 |
| P2_A5F_H13_pG | CRGLGLALVRRpGRRGLGLFLVR​C |  | 12.50 |
| P2_A5F_H13_GV | CRGLGLALVRRGVRRGLGLFLVR​C |  | 50.00 |
| P2_A5F_H13_GV | CRGLGLALVRRGVRRGLGLFLVR​C |  | 25.00 |
| P2_H12_GV_RR | RRGLGLALVCRRGVCRRGLGLALVRR | Increased arginine  & β-turn-promoting linker &  cysteine | 3.13 |
| V7Y_H12_pG_M | RMGLGLALYCRRpGRRCGLGLALYR | Increased interaction affinity  & β-turn-promoting linker  &  cysteine | 3.13 |
| V7Y_H12_pG_M_RR | RRMGLGLALYCRRpGRRCGLGLALYRR | Increased aggregation propensity & Increased interaction affinity  & β-turn-promoting linker &  cysteine | 3.13 |
