# Supplementary material for: Enhanced therapeutic window for antimicrobial Pept-ins by investigating their structure-activity relationship
Source: PLoS One. 2023 Mar 31;18(3):e0283674. doi: 10.1371/journal.pone.0283674 (PMC10065276; doi:10.1371/journal.pone.0283674)
Supplement: S10 Table — (DOCX) [file pone.0283674.s016.docx]

**S10 Table. MIC of P2 variants (4 arginine and linker modification)**

| **Name** | **Sequence** | **BL21 MIC (μg/mL)** | **Comment** |
| --- | --- | --- | --- |
| P2 | RGLGLALVRRPRGLGLALVRR | 12.50 |  |
| P2_4R | RGLGLALVRAPRGLGLALVAR | 25.00 | 4 ariginine |
| P2_4R_PEG | RGLGLALVRA(PEG)RGLGLALVAR | 50.00 | Flexible linker |
| P2_4R_pP | RGLGLALVRApPRGLGLALVAR | 50.00 | β-turn-promoting linker |
| P2_4R_fP | RGLGLALVRAfPRGLGLALVAR | 25.00 |  |
| P2_4R_GV | RGLGLALVRAGVRGLGLALVAR | 6.25 |  |
| P2_4R_L | RGLGLALVRALRGLGLALVAR | 12.50 | Helix-promoting linker |
| P2_4R_I | RGLGLALVRAIRGLGLALVAR | 12.50 |  |
| P2_4R_M | RGLGLALVRAMRGLGLALVAR | 12.50 |  |
| P2_4R_V | RGLGLALVRAVRGLGLALVAR | 12.50 |  |
| P2_4R_F | RGLGLALVRAFRGLGLALVAR | 12.50 |  |
| P2_4R_W | RGLGLALVRAWRGLGLALVAR | 12.50 |  |
| P2_4R_A | RGLGLALVRAARGLGLALVAR | 12.50 |  |
| P2_4R_R | RGLGLALVRARRGLGLALVAR | 12.50 |  |
