# Supplementary material for: Enhanced therapeutic window for antimicrobial Pept-ins by investigating their structure-activity relationship
Source: PLoS One. 2023 Mar 31;18(3):e0283674. doi: 10.1371/journal.pone.0283674 (PMC10065276; doi:10.1371/journal.pone.0283674)
Supplement: S11 Table — (DOCX) [file pone.0283674.s017.docx]

**S11 Table. MIC of P2 variants against P2-resistant strain strain (E. coli XL1 Red)**

|  |  | **MIC (μg/mL)** | | **Comment** |
| --- | --- | --- | --- | --- |
| **Name** | **Sequence** | **Ancestor** | **P2-Resistant cells** |  |
| P2 | RGLGLALVRRPRGLGLALVRR | 6.25 | 200.00 |  |
| P2 (8R)_Rigid linker | RGLGLALVRRPRPRPRGLGLALVRR | 6.25 | 50.00 | Linker modification |
| HS231_P2 _pG linker | RGLGLALVRRpGRGLGLALVRR | 3.13 | 50.00 |  |
| HS231_P2_ fP linker | RGLGLALVRRfPRGLGLALVRR | 3.13 | 25.00 |  |
| P2_H6 | RCLGLALVRRVRGLGLALRRGSC | 3.13 | 25.00 | Disulphide bond formation |
| P2_H12 | RGLGLALVCRRGVCRGLGLALVRR | 3.13 | 6.25 |  |
| P2_M | RMGLGLALVRRPRGLGLALVRR | 6.25 | 50.00 | Increased aggregation propensity |
| P2_Y | RYGLGLALVRRPRGLGLALVRR | 6.25 | 50.00 |  |
| P2_L | RLGLGLALVRRPRGLGLALVRR | 6.25 | 50.00 |  |
| P2_F | RFGLGLALVRRPRGLGLALVRR | 3.13 | 25.00 |  |
| P2_TY | RTYGLGLALVRRPRGLGLALVRR | 3.13 | 25.00 |  |
| P2_TF | RTFGLGLALVRRPRGLGLALVRR | 6.25 | 50.00 |  |
| P2_TTY | RTTYGLGLALVRRPRGLGLALVRR | 3.13 | 25.00 |  |
| P2_TTL | RTTLGLGLALVRRPRGLGLALVRR | 6.25 | 50.00 |  |
| P2_TTF | RTTFGLGLALVRRPRGLGLALVRR | 3.13 | 25.00 |  |
| P2_IM | RIMGLGLALVRRPRGLGLALVRR | 3.13 | 25.00 |  |
| P2_LI | RLIGLGLALVRRPRGLGLALVRR | 3.13 | 12.50 |  |
| P2_VFV | RVFVGLGLALVRRPRGLGLALVRR | 3.13 | 6.25 |  |
