# Supplementary material for: Enhanced therapeutic window for antimicrobial Pept-ins by investigating their structure-activity relationship
Source: PLoS One. 2023 Mar 31;18(3):e0283674. doi: 10.1371/journal.pone.0283674 (PMC10065276; doi:10.1371/journal.pone.0283674)
Supplement: S12 Table — (DOCX) [file pone.0283674.s018.docx]

**S12 Table. MIC of P2 variants against clinically isolated multi-drug resistant isolates.**

| **Organism** | **MIC (μg/mL)** | | | | | | | |
| --- | --- | --- | --- | --- | --- | --- | --- | --- |
|  | **P2** | **P2_4R_L** | **P2_4R_GV** | **P2_A5F** | **V7Y(P2)** | **P2_H12_PEG** | **P2_H12_GV** | **Tobramycin** |
| *E. coli* | 32 | 16 | 32 | 8 | 8 | 8 | 8 | 0.5 |
| *E. coli* | 32 | 16 | 32 | 8 | 8 | 8 | 8 | >1 |
| *E. coli* | 32 | 16 | 32 | 8 | 8 | 8 | 16 | 1 |
| *E. coli* | 32 | 16 | 32 | 8 | 8 | 8 | 8 | 1 |
| *E. coli* | 32 | 32 | 32 | 8 | 8 | 8 | 16 | >1 |
| *E. coli* | 32 | 16 | 32 | 8 | 8 | 8 | 16 | >1 |
| *E. coli* | 32 | 16 | 16 | 8 | 8 | 8 | 8 | 1 |
| *E. coli* | 32 | 16 | 32 | 8 | 8 | 8 | 16 | 0.5 |
| *E. coli* | 32 | 16 | 16 | 8 | 8 | 8 | 16 | >1 |
| *E. coli* | 32 | 16 | 32 | 8 | 8 | 8 | 8 | 1 |
| *E. coli* | 32 | 16 | 32 | 8 | 8 | 8 | 8 | 1 |
| *E. coli* | 32 | 16 | 32 | 8 | 8 | 8 | 16 | 1 |
| *E. coli* | 32 | 16 | 32 | 8 | 8 | 8 | 16 | >1 |
| *E. coli* | 16 | 16 | 32 | 8 | 8 | 8 | 8 | 1 |
| *E. coli* | >32 | >32 | >32 | 16 | 16 | 16 | 16 | 0.5 |
| *E. coli* | 32 | 16 | 32 | 8 | 8 | 8 | 8 | >1 |
| *E. coli* | 16 | 8 | 16 | ≤4 | 8 | ≤4 | 8 | >1 |
| *E. coli* | 32 | 16 | 32 | 8 | 8 | 8 | 16 | >1 |
| *E. coli* | 32 | 16 | 16 | 8 | 8 | 8 | 8 | 1 |
| *E. coli* | 32 | 16 | 32 | 8 | 8 | 8 | 8 | >1 |
| *E. coli* | 16 | 16 | 16 | 8 | 8 | 8 | 8 | >1 |
| *E. coli* | 32 | 16 | 16 | 8 | 8 | 8 | 16 | 1 |
| *E. coli* | 32 | 16 | 32 | 8 | 8 | 8 | 8 | 0.5 |
| *E. coli* | 32 | 16 | 32 | 8 | 8 | 8 | 16 | >1 |
| *E. coli* | 32 | 32 | 32 | 8 | 8 | 16 | 16 | 0.5 |
| *E. coli* | 32 | 16 | 32 | 8 | 8 | 8 | 16 | 0.5 |
| *E. coli* | 32 | 16 | 32 | 8 | 8 | 8 | 8 | 0.5 |
| *E. coli* | 32 | 32 | 32 | 8 | 16 | 8 | 16 | >1 |
| *E. coli* | 32 | 16 | 16 | 8 | 8 | 8 | 8 | >1 |
| *E. coli* | 32 | 16 | 32 | 8 | 8 | 8 | 16 | >1 |
| *E. coli* | 32 | 16 | 32 | 8 | 8 | 8 | 8 | >1 |
| *E. coli* | 32 | 16 | 32 | 8 | 8 | 8 | 16 | 0.5 |
| *E. coli* | 32 | 16 | 32 | 8 | 8 | 8 | 8 | >1 |
| *E. coli* | 32 | 16 | 32 | 8 | 8 | 8 | 16 | >1 |
| *A. baumannii* | 32 | 8 | 16 | 8 | 8 | 8 | 16 | >1 |
| *A. baumannii* | 16 | 8 | 8 | 8 | 8 | 8 | 8 | >1 |
| *A. baumannii* | 16 | 16 | 32 | 8 | 8 | 8 | 8 | >1 |
| *A. baumannii* | 32 | 8 | 16 | 8 | 8 | 8 | 16 | >1 |
| *A. baumannii* | 16 | 16 | 32 | 8 | 8 | 8 | 8 | 0.25 |
| *A. baumannii* | 16 | 8 | 16 | 8 | 8 | 8 | 16 | 0.5 |
| *A. baumannii* | 16 | 8 | 16 | 8 | 8 | 8 | 8 | >1 |
| *A. baumannii* | 16 | 8 | 16 | 8 | 8 | 8 | 16 | >1 |
| *A. baumannii* | 32 | 16 | 32 | 8 | 16 | 8 | 8 | >1 |
| *A. baumannii* | 16 | 16 | 16 | 8 | 8 | 8 | 8 | >1 |
| *A. baumannii* | 16 | 8 | 16 | 8 | 8 | 8 | 16 | >1 |
| *A. baumannii* | 16 | 8 | 16 | 8 | 8 | 8 | 8 | 1 |
| *K. pneumoniae* | >32 | >32 | >32 | 16 | 16 | 32 | 32 | >1 |
| *K. pneumoniae* | >32 | >32 | >32 | 16 | 32 | 16 | 32 | 0.25 |
| *K. pneumoniae* | >32 | >32 | >32 | >32 | >32 | 32 | >32 | 0.5 |
| *K. pneumoniae* | 16 | 32 | 32 | 8 | 8 | 8 | 8 | >1 |
| *K. pneumoniae* | >32 | >32 | >32 | 32 | 32 | 32 | 32 | 1 |
| *K. pneumoniae* | >32 | >32 | >32 | 32 | >32 | 32 | >32 | >1 |
| *K. pneumoniae* | 32 | 8 | 32 | 8 | 8 | 8 | 8 | 1 |
| *K. pneumoniae* | >32 | >32 | >32 | >32 | >32 | >32 | >32 | >1 |
| *K. pneumoniae* | >32 | >32 | >32 | 32 | 32 | 16 | 32 | >1 |
| *K. pneumoniae* | >32 | >32 | >32 | 32 | >32 | >32 | 32 | >1 |
| *K. pneumoniae* | >32 | >32 | >32 | 32 | 32 | 32 | 32 | >1 |
| *K. pneumoniae* | >32 | >32 | >32 | 16 | 16 | 16 | 32 | 0.5 |
