# Supplementary material for: Enhanced therapeutic window for antimicrobial Pept-ins by investigating their structure-activity relationship
Source: PLoS One. 2023 Mar 31;18(3):e0283674. doi: 10.1371/journal.pone.0283674 (PMC10065276; doi:10.1371/journal.pone.0283674)
Supplement: S13 Table — (DOCX) [file pone.0283674.s019.docx]

**S13 Table. Proteins identified in *K. pneumoniae* and *A. baumannii* which contain the APR of P2 (GLGLALV) by allowing 1 mismatch**

| APR | Protein name | Number of mutation | Strain |
| --- | --- | --- | --- |
| GLGLALV | APR of P2 |  |  |
| GIGLILS | ACIBA Major facilitator superfamily MFS | 1 | *A. baumannii* |
| GLGLLLV | MFS family transporter | 1 |  |
| GLGLAVV | Histidine kinase | 1 |  |
| GLGLAIV | Histidine kinase | 1 |  |
| GLGLALS | Histidine kinase | 1 |  |
| GLGLAIV | Phosphate regulon sensor protein PhoR | 1 |  |
| PLGLALV | DMT superfamily metabolite efflux protein | 1 |  |
| GIGLALV | Oxygenase subunit | 1 |  |
| GLGLALW | NADH dehydrogenase I subunit M | 1 |  |
| FLGLALV | DUF333 domain-containing protein | 1 |  |
| LLGLALV | Lipid III flippase | 1 | *k. pneumoniae* |
| ELGLALV | Multidrug resistance protein B | 1 |  |
| GLGLLLV | Nickel transport ATP-binding protein | 1 |  |
| GLGLALI | Cystathionine beta-synthase | 1 |  |
| GLGLAMV | Outer membrane protein OmpH | 1 |  |
| GLGLAFV | Sensor protein creC | 1 |  |
| GLGLAIV | Osmolarity sensor protein envZ | 1 |  |
| GRGLALV | Uncharacterized protein | 1 |  |
| GLGLAIV | Phosphate regulon sensor protein phoR | 1 |  |
| GLGLAHV | M20_dimer domain-containing protein | 1 |  |
| GLGLGLV | Cysteine Degradation Operon Regulator | 1 |  |
| GLSLALV | Putative cystathionine beta-synthase domain protein | 1 |  |
| GLPLALV | Phosphoglycolate phosphatase | 1 |  |
| GLGLAMV | Dipeptide and tripeptide permease A | 1 |  |
| GLLLALV | Formate hydrogenlyase subunit | 1 |  |
| GLGLAIV | Sensor protein cpxA | 1 |  |
| GLGLAVV | Sensor protein hydH | 1 |  |
| GLGIALV | Transcriptional regulator, LysR family protein | 1 |  |
| GLGLAIV | Sensor protein rstB | 1 |  |
| GLGLAYV | Transcriptional regulator, LysR family protein | 1 |  |
| GLALALV | Na+/H+ antiporter nhaP | 1 |  |
