# Supplementary material for: Enhanced therapeutic window for antimicrobial Pept-ins by investigating their structure-activity relationship
Source: PLoS One. 2023 Mar 31;18(3):e0283674. doi: 10.1371/journal.pone.0283674 (PMC10065276; doi:10.1371/journal.pone.0283674)
Supplement: S14 Table — (DOCX) [file pone.0283674.s020.docx]

**S14 Table. MIC of P2 variants used for toxicity study**

| **Name** | **Sequence** | **BL21 MIC (μg/mL)** | **Comment** |
| --- | --- | --- | --- |
| P2 | RGLGLALVRRPRGLGLALVRR | 12.50 |  |
| P2_VFV | RVFVGLGLALVRRPRGLGLALVRR | 12.50 | Increased aggregation propensity |
| P2_IM | RIMGLGLALVRRPRGLGLALVRR | 6.25 |  |
| P2_M | RMGLGLALVRRPRGLGLALVRR | 6.25 |  |
| P2_A5F | RGLGLALVRRPRGLGLFLVRR​ | 3.13 | Aggregate core structure optimization |
| V7Y (P2) | RGLGLALYRRPRGLGLALYRR​ | 3.13 |  |
| P2_pG | RGLGLALVRRpGRGLGLALVRR | 6.25 | Linker modification or disuphide bond formation |
| P2_GV | RGLGLALVRRGVRGLGLALVRR | 6.25 |  |
| P2_H12_GV | GLGLALVCRRGVCRGLGLALVRR | 3.13 |  |
| P2_H12_PEG | RGLGLALVCRR(PEG)CRGLGLALVRR | 3.13 |  |
| P2_4R | RGLGLALVRAPRGLGLALVAR | 25.00 | Arginine modification |
| P2_4R_GV | RGLGLALVRAGVRGLGLALVAR | 6.25 |  |
| P2_4R_L | RGLGLALVRALRGLGLALVAR | 12.50 |  |
| P2_RR | RRGLGLALVRRPRRGLGLALVRR | 3.13 |  |
