# Supplementary material for: Enhanced therapeutic window for antimicrobial Pept-ins by investigating their structure-activity relationship
Source: PLoS One. 2023 Mar 31;18(3):e0283674. doi: 10.1371/journal.pone.0283674 (PMC10065276; doi:10.1371/journal.pone.0283674)
Supplement: Supplementary file 16 [file pone.0283674.s002.docx]

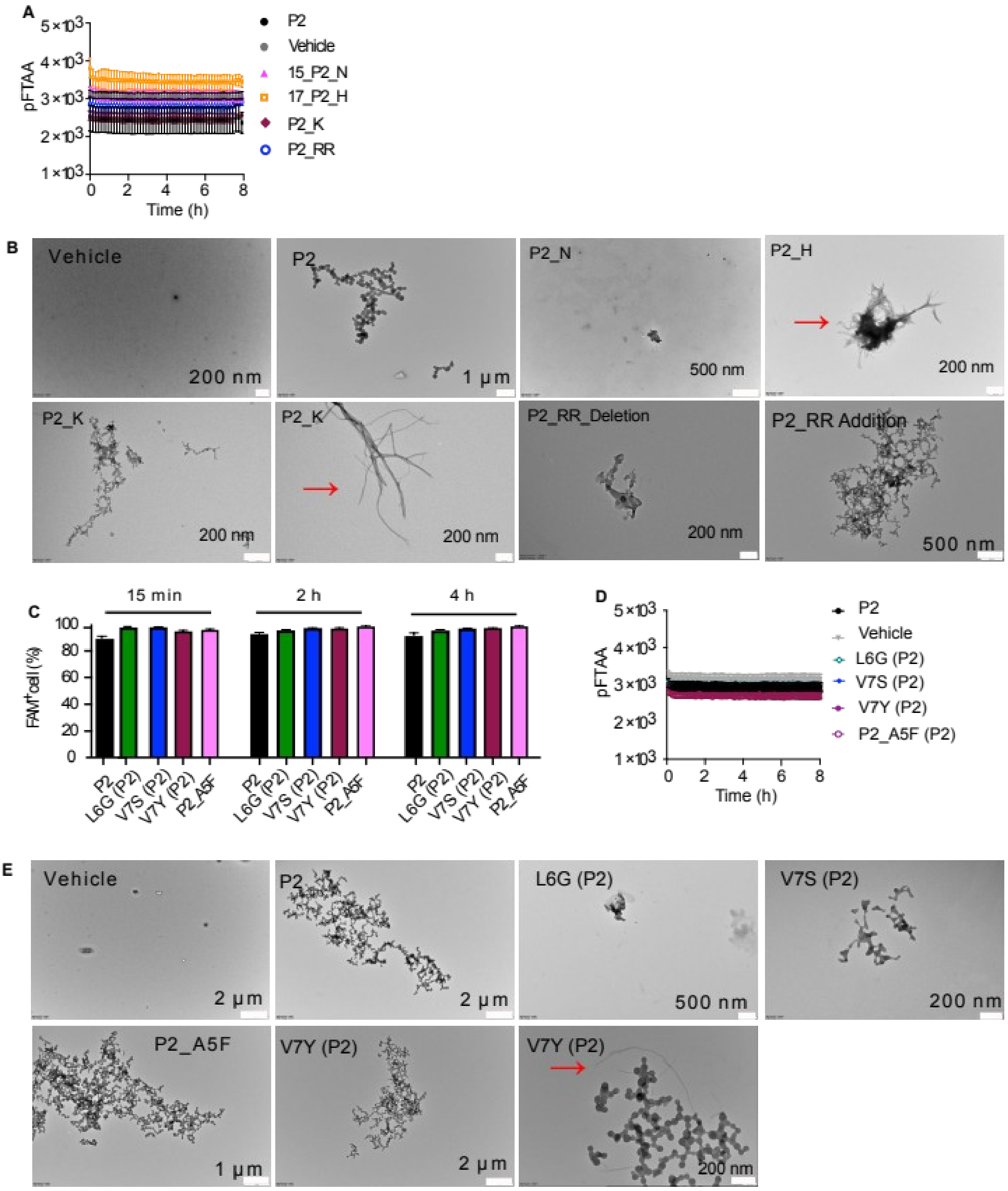
A
5×10^3
4×10^3
3×10^3
2×10^3
1×10^3
pFTAA
0
2
4
6
8
Time (h)
P2
Vehicle
15_P2_N
17_P2_H
P2_K
P2_RR
B
Vehicle
200 nm
P2
1 μm
P2_N
500 nm
P2_H
200 nm
P2_K
200 nm
P2_K
200 nm
P2_RR_Deletion
200 nm
P2_RR Addition
500 nm
C
15 min
2 h
4 h
FAM+cell (%)
100
80
60
40
20
0
P2
L6G (P2)
V7S (P2)
V7Y (P2)
P2_A5F
D
5×10^3
4×10^3
3×10^3
2×10^3
1×10^3
pFTAA
0
2
4
6
8
Time (h)
P2
Vehicle
L6G (P2)
V7S (P2)
V7Y (P2)
P2_A5F (P2)
E
Vehicle
2 μm
P2
2 μm
L6G (P2)
500 nm
V7S (P2)
200 nm
P2_A5F
1 μm
V7Y (P2)
2 μm
V7Y (P2)
200 nm
