# Supplementary material for: Enhanced therapeutic window for antimicrobial Pept-ins by investigating their structure-activity relationship
Source: PLoS One. 2023 Mar 31;18(3):e0283674. doi: 10.1371/journal.pone.0283674 (PMC10065276; doi:10.1371/journal.pone.0283674)
Supplement: Supplementary file 17 [file pone.0283674.s003.docx]

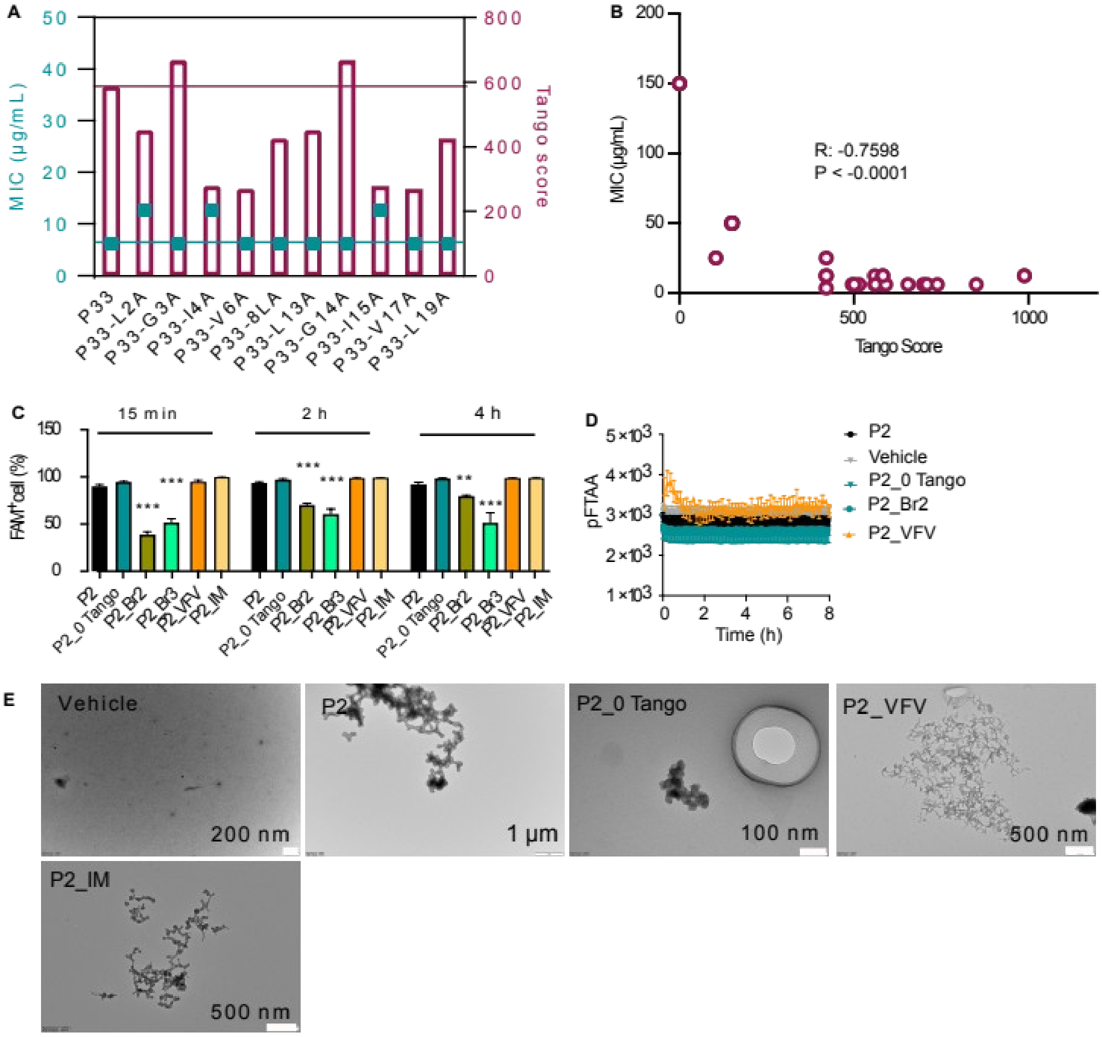
A
MIC (μg/mL)
Tango score
0
10
20
30
40
50
0
200
400
600
800
P33
P33-L2A
P33-G3A
P33-I4A
P33-V6A
P33-8LA
P33-L13A
P33-G14A
P33-I15A
P33-V17A
P33-L19A
B
MIC (μg/mL)
R: -0.7598
P < -0.0001
Tango Score
0
50
100
150
200
0
500
1000
C
15 min
2 h
4 h
FAM+cell (%)
0
50
100
150
P2
P2_0 Tango
P2_Br2
P2_Br3
P2_VFV
P2_IM
D
pFTAA
Time (h)
P2
Vehicle
P2_0 Tango
P2_Br2
P2_VFV
E
Vehicle
200 nm
P2
1 μm
P2_0 Tango
100 nm
P2_VFV
500 nm
P2_IM
500 nm
