# Supplementary material for: Enhanced therapeutic window for antimicrobial Pept-ins by investigating their structure-activity relationship
Source: PLoS One. 2023 Mar 31;18(3):e0283674. doi: 10.1371/journal.pone.0283674 (PMC10065276; doi:10.1371/journal.pone.0283674)
Supplement: Supplementary file 18 [file pone.0283674.s005.docx]

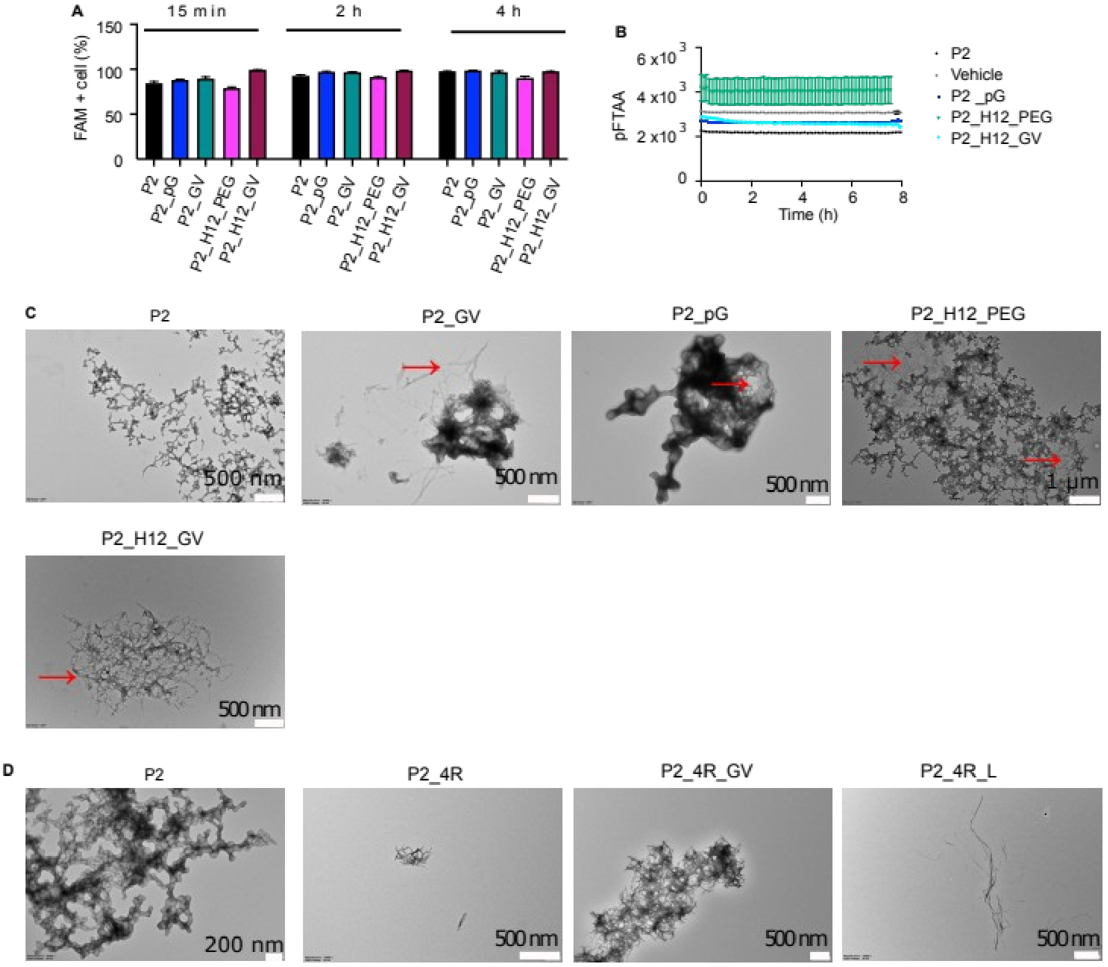
A
15 min
2 h
4 h
FAM + cell (%)
150
100
50
0
P2
P2_pG
P2_GV
P2_H12_PEG
P2_H12_GV
B
pFTAA
6 x10 3
4 x10 3
2 x10 3
0
0
2
4
6
8
Time (h)
P2
Vehicle
P2 _pG
P2_H12_PEG
P2_H12_GV
C
P2
500 nm
P2_GV
500 nm
P2_pG
500 nm
P2_H12_PEG
1 μm
P2_H12_GV
500 nm
D
P2
200 nm
P2_4R
500 nm
P2_4R_GV
500 nm
P2_4R_L
500 nm
